# Potential impacts of synthetic food dyes on activity and attention in children: a review of the human and animal evidence

**DOI:** 10.1186/s12940-022-00849-9

**Published:** 2022-04-29

**Authors:** Mark D. Miller, Craig Steinmaus, Mari S. Golub, Rosemary Castorina, Ruwan Thilakartne, Asa Bradman, Melanie A. Marty

**Affiliations:** 1 Office of Environmental Health Hazard Assessment, California Environmental Protection Agency, 1515 Clay St, Oakland CA, and 1001 I St, Sacramento, California, USA; 2 Center for Environmental Research and Community Health, School of Public Health, University of California, 2121 Berkeley Way, Berkeley, California, USA; 3 Department of Public Health, School of Social Sciences, Humanities and Arts, University of California, Merced, 5200 N Lake Road, Merced, CA USA

**Keywords:** Synthetic food dyes, Children, Behavior, Clinical trials, Animal toxicology

## Abstract

Concern that synthetic food dyes may impact behavior in children prompted a review by the California Office of Environmental Health Hazard Assessment (OEHHA). OEHHA conducted a systematic review of the epidemiologic research on synthetic food dyes and neurobehavioral outcomes in children with or without identified behavioral disorders (particularly attention and activity). We also conducted a search of the animal toxicology literature to identify studies of neurobehavioral effects in laboratory animals exposed to synthetic food dyes. Finally, we conducted a hazard characterization of the potential neurobehavioral impacts of food dye consumption. We identified 27 clinical trials of children exposed to synthetic food dyes in this review, of which 25 were challenge studies. All studies used a cross-over design and most were double blinded and the cross-over design was randomized. Sixteen (64%) out of 25 challenge studies identified some evidence of a positive association, and in 13 (52%) the association was statistically significant. These studies support a relationship between food dye exposure and adverse behavioral outcomes in children. Animal toxicology literature provides additional support for effects on behavior. Together, the human clinical trials and animal toxicology literature support an association between synthetic food dyes and behavioral impacts in children. The current Food and Drug Administration (FDA) acceptable daily intakes are based on older studies that were not designed to assess the types of behavioral effects observed in children. For four dyes where adequate dose-response data from animal and human studies were available, comparisons of the effective doses in studies that measured behavioral or brain effects following exposure to synthetic food dyes indicate that the basis of the ADIs may not be adequate to protect neurobehavior in susceptible children. There is a need to re-evaluate exposure in children and for additional research to provide a more complete database for establishing ADIs protective of neurobehavioral effects.

**Supplementary Information:**

The online version contains supplementary material available at 10.1186/s12940-022-00849-9.

## Background

Concerns about possible associations between exposure to synthetic food dyes and the exacerbation of symptoms of Attention Deficit/Hyperactivity Disorder (ADHD) in children have surfaced periodically since the 1970s. The concern prompted the California legislature to request a review by the California Environmental Protection Agency’s Office of Environmental Health Hazard Assessment (OEHHA) of available studies to evaluate whether the synthetic food dyes currently allowed in foods and medications in the United States impact neurobehavior in children [1]. This paper provides an overview of key portions of OEHHA’s peer-reviewed assessment, specifically the evaluation of the clinical trials of synthetic food dyes in children and available animal toxicology studies, as well as discussion of our hazard characterization and the possible public health implications of our findings.

Our evaluation focused on seven of the nine food dyes subject to FD&C batch certification by the US Food and Drug Administration (FDA) and approved for general use in food in the US (Table 1). These seven dyes contribute nearly all of the exposure to synthetic food dyes for the general US public [1]. The term “FD&C batch-certified” refers to the Food Drug and Cosmetic Act requirements for chemical analysis of each manufactured batch of food dye to ensure that specific contaminants are present below legal limits. OEHHA evaluated the literature to determine whether there is any evidence supporting the association of exposure to synthetic food dyes with adverse neurobehavioral impacts in children in the general population with or without a diagnosis of ADHD.

**Table 1:** US FDA batch-certified food colors addressed in OEHHA’s report

Food Dye	Common Synonym	CAS #
FD&C Blue No. 1	Brilliant Blue	3844-45-9
FD&C Blue No. 2	Indigo Carmine, Indigotine	860–22-0
FD&C Green No. 3	Fast Green	2353-45-9
FD&C Red No. 3	Erythrosine	16,423–68-0
FD&C Red No. 40	Allura Red	25,956–17-6
FD&C Yellow No. 5	Tartrazine	1934-21-0
FD&C Yellow No. 6	Sunset Yellow	2783-94-0

## Methods

The literature review methods were designed to identify all the literature most relevant to the assessment of evidence on the neurological or neurobehavioral effects of the synthetic food dyes listed in Table 1. The search was executed to identify peer-reviewed open-source and proprietary journal articles, print and digital books, reports, and gray literature that potentially reported relevant toxicological and epidemiological information. We also included Citrus Red No. 2 and Orange B/CI Acid Orange in the search terms since these food dyes are part of an overlapping literature that might contain information on the commonly used FD&C synthetic food dyes. PubMed MeSH browser (PubMed MeSH browser) and PubChem (PubChem) were used to identify subject headings, other index terms and synonyms for the food dyes of interest and their metabolites, as well as for the concepts related to exposure, food, mechanisms of action, and neurological outcomes. Preliminary searches were run and results reviewed to identify additional terms. The concepts were combined in the following manner:

### ((food/dietary terms) AND (specific food dye terms)) OR ((specific food dye terms) AND (neurological outcome terms) OR (general exposure terms) OR (mechanisms of action terms))

The detailed search strategy executed in PubMed on November 26, 2018 is summarized in the additional information (Table A.1). This search was run again to capture literature updates, on March 8, 2019 and April 22, 2019, and again in October 2020.

Additional databases (PubMed, Embase, Scopus) and other data sources (European Food Safety Authority (EFSA) Journal, EFSA Scientific Output, US FDA Safety Information Office, University of California, San Francisco Food Industry Documents Archive, and Dyes and Pigments Journal) were also searched; strategies were tailored according to the search features unique to each database and data source. Relevant literature was also identified from citations in individual articles. In addition, we searched NIH RePort to identify additional unpublished clinical trials or animal research. In our systematic review of the epidemiologic research on synthetic food dyes and neurobehavioral outcomes in children, we summarized the major strengths and weaknesses of each study, described any consistencies across study results, and if heterogeneity exists, identified its sources as far as possible [1].

Our epidemiologic review focused on clinical trials. A major advantage of this type of study is that investigators generally have control over the exposure which can help reduce bias and confounding compared to other study designs. Next, we conducted systematic evaluations of study methods and quality to ensure an emphasis on the high quality studies for our conclusions. In evaluating study quality, we utilized criteria based on the National Toxicology Program’s OHAT Risk of Bias Rating Tool [2]. We modified these to be specific to randomized clinical trials (RCT) on artificial food dyes and childhood neurobehavior. We examined several key characteristics of each study to assess study quality including design, participant selection, exposure levels, age groups, washout period, infractions, outcome metric, and funding (Table A.2). This table also includes key information on results including statistical significance, effect size, dose-response, and subgroups. The coding used in our statistical analyses and quality scoring is provided in Tables A.3 and A.4. These tables show the criteria used to evaluate study quality, which included randomization, placebo use, dropout rate, blinding, whether dose-response was assessed, outcome metric validation, replication, and adequate washout. All this information was considered in making our overall conclusions about the human study results.

In determining whether the study reported an association, we define association as either a statistically significant outcome (*p* value <.05 or 95% confidence intervals that excluded 1.0 for relative risk estimates or 0 for mean differences) or an effect size ≥20% or standardized effect size ≥0.20. Most studies involved small sample sizes and thus may not have had sufficient statistical power to identify effects that are relatively small but still of public health importance. Because of this, in addition to statistical significance, bias and effect size were also considered in our evaluations of association and causal inference. There are several arguments against solely using statistical significance to identify associations [3, 4].

We searched the animal toxicology literature and identified numerous studies of neurobehavioral effects in laboratory animals exposed to synthetic food dyes. These included studies of exposures during prenatal, infant, and juvenile development, examining neurobehavioral effects in the offspring manifest during development and/or later in adult animals. The availability of studies at different developmental stages allowed a comprehensive review of adverse developmental effects, although it limited the ability to compare results across study designs, as exposures during different developmental stages may manifest differently later in life. The OEHHA report reviewed all available studies and provided strengths and limitations for the individual studies [1].

Acceptable Daily Intakes (ADIs) for synthetic food dyes were established by the US FDA between the 1960s and the1980s based on general toxicology studies. OEHHA therefore also evaluated whether newer studies that included neurobehavioral assessment would be useful for developing updated acceptable exposure levels that explicitly account for and protect against neurobehavioral effects of individual food dyes. OEHHA compared the results of those specific studies to the existing US FDA ADIs, as well as ADIs developed by the Joint FAO/WHO Expert Committee on Food Additives (JECFA).

## Results

### Review of clinical trial studies

In total, 27 clinical trials were identified that met each of the following criteria:

1. Human study
2. Clinical trial design
3. Participants were given a known quantity of synthetic food dyes or a diet low in or eliminating synthetic food dyes
4. A neurobehavioral outcome related to hyperactivity or inattention was assessed
5. The majority of participants were children ≤19 years of age
6. The effects of an active ingredient or elimination diet were compared to those of a placebo

Studies were excluded if they were:

1. Studies involving cohort, case-control, or cross-sectional designs
2. Studies that assessed the effects of a broad range of food groups, including elimination studies, and did not specifically evaluate synthetic food dyes. Any effect identified in such studies would be difficult to ascribe specifically to synthetic food dyes.

No exclusions were made based on the number of participants, participation rates, blinding, randomization, or source (e.g., government reports), although each of these factors was considered in our review of study quality and in our overall conclusions.

**Fig. 1:**
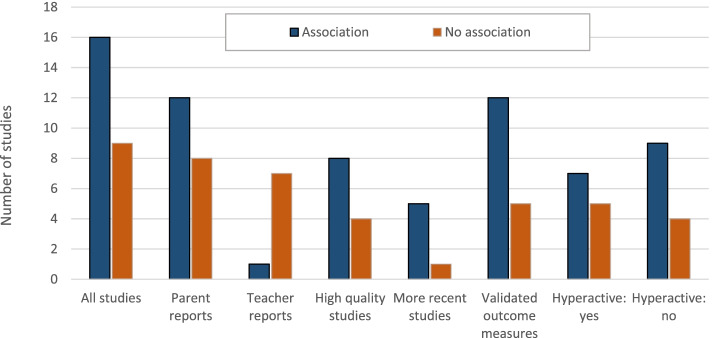
Number of clinical studies reporting positive associations by key study variables

Figure 1 presents the results of our literature search as the number of clinical studies reporting adverse neurobehavioral outcomes by key study variables. Of the 27 studies meeting our criteria for inclusion, 25 involved challenge studies, which we consider most relevant as they directly challenge children with food dyes, and two involved diet elimination studies. Detailed descriptions of the 25 included challenge studies are provided in Table A.2. Table 2 below summarizes the characteristics and overall findings of the reviewed challenge studies. Several studies of exposure to dye mixtures also included other dyes not used in the US.

**Table 2:** Clinical trials of synthetic food dyes and neurobehavioral outcomes in children: summary of study results

	Total	No association^**a**^		Association identified^**b**^		Large effect size^**b**^		Statistically significant^**b**^		***p***^**c**^
Variable	N	N	%	N	%	N	%	N	%	
All studies	25	9	36.0	16	64.0	3	12.0	13	52.0	
Group results^d^										
Parent	14	7	50.0	7	50.0	1	7.1	6	42.9	Ref
Teacher	7	6	85.7	1	14.3	0	0.0	1	14.3	0.11
Other	14	9	64.3	5	35.7	2	14.3	3	21.4	0.45
Individual results^e^										
Parent	12	3	25.0	9	75.0	4	33.3	5	41.7	Ref
Teacher	2	1	50.0	1	50.0	1	50.0	0	0.0	0.12
Other	5	1	20.0	4	80.0	3	60.0	1	20.0	0.91
Study quality^f^										
Higher	12	4	33.3	8	66.7	2	16.7	6	50.0	Ref
Lower	13	5	38.5	8	61.5	1	7.7	7	53.8	0.79
Publication year										
Before 1990	19	8	42.1	11	57.9	3	15.8	8	42.1	Ref
1990 and later	6	1	16.7	5	83.3	0	0.0	5	83.3	0.26
Location										
United States	10	4	40.0	6	60.0	2	20.0	4	40.0	Ref
Elsewhere	15	5	33.3	10	66.7	1	6.7	9	60.0	0.73
In hyperactive only^g^										
Yes	12	5	41.7	7	58.3	1	8.3	6	50.0	Ref
No	13	4	30.8	9	69.2	2	15.4	7	53.8	0.57
Prior responders only^h^										
Yes	14	7	50.0	7	50.0	1	7.1	6	42.9	Ref
No	11	2	18.2	9	81.8	2	18.2	7	63.6	0.10
No. of participants										
< 20	15	7	46.7	8	53.3	2	13.3	6	40.0	Ref
20–100	7	1	14.3	6	85.7	1	14.3	5	71.4	0.14
≥ 100	3	1	33.3	2	66.7	0	0.0	2	66.7	0.67
RCDP										
Yes	16	6	37.5	10	62.5	2	12.5	8	50.0	Ref
No	9	3	33.3	6	66.7	1	11.1	5	55.6	0.83
Challenge agents										
Multiple dyes	19	7	36.8	12	63.2	3	15.8	9	47.4	Ref
Tartrazine only	6	2	33.3	4	66.7	0	0.0	4	66.7	0.88
Daily dose (mg)										
≤ 10	4	2	50.0	2	50.0	0	0.0	2	50.0	Ref
11–35	7	4	57.1	3	42.9	0	0.0	3	42.9	0.82
36–99	8	2	25.0	6	75.0	2	25.0	4	50.0	0.39
≥ 100+	3	1	33.3	2	66.7	0	0.0	2	66.7	0.66
Unclear	3	0	0.0	3	100.0	1	33.3	2	66.7	0.15
Washout > 2 days										
Yes	11	6	54.5	5	45.5	1	9.1	4	36.4	Ref
No	14	3	21.4	11	78.6	2	14.3	9	64.3	0.09
Food dyes only										
Yes	22	9	40.9	13	59.1	3	13.6	10	45.5	Ref
Additional agent^i^	3	0	0.0	3	100.0	0	0.0	3	100.0	0.17
Validated										
Yes	17	5	29.4	12	70.6	2	11.8	10	58.8	Ref
No	8	4	50.0	4	50.0	1	12.5	3	37.5	0.32
Outcome timing										
Hourly	9	4	44.4	5	55.6	2	22.2	3	33.3	Ref
Daily	6	0	0.0	6	100.0	0	0.0	6	100.0	0.06
Several per week	3	1	33.3	2	66.7	1	33.3	1	33.3	0.74
Weekly	3	1	33.3	2	66.7	0	0.0	2	66.7	0.74
Greater than weekly	1	0	0.0	1	100.0	0	0.0	1	100.0	0.39
Unclear	3	3	100.0	0	0.0	0	0.0	0	0.0	0.09
Full results										
Yes	12	5	41.7	7	58.3	2	16.7	5	41.7	Ref
No	13	4	30.8	9	69.2	1	7.7	8	61.5	0.57
Low infractions^j^										
Yes	16	7	43.8	9	56.3	3	18.8	6	37.5	Ref
No or unknown	9	2	22.2	7	77.8	0	0.0	7	77.8	0.28

*Abbreviations: RCDP*, studies that are randomized crossover design, double blinded, and placebo controlled; Ref, reference category

Only includes studies involving an active challenge i.e. diet elimination trials were not included in this table

^a^ Studies that did not report an association that was statistically significant, an effect size ≥20%, or standardized effect size ≥0.20

^b^ Studies that reported a statistically significant association, an effect size ≥20%, or standardized effect size ≥0.20. This category combines the studies listed under the “Large effect size” and “Statistically significant” columns. The “Statistically significant” column includes any study reporting a statistically significant association, regardless of effect size. The “Large effect size” column includes studies that reported an effect size ≥20% or a standardized effect size ≥0.20 but the results were not statistically significant

^c^ Chi-square *p*-value comparing proportion of studies finding no association (i.e. those in the “No association” column) to the proportion of studies finding an association (i.e. those in the “Association identified” column)

^d^ In studies that presented group means, provides results by the source of the outcome information (Parent, Teacher, or Other). The number of studies listed here is greater than the total number of studies since several studies presented results for more than one outcome source

^e^ In studies that presented results for individual participants, provides results by the source of the outcome information (Parent, Teacher, or Other). Several studies presented results for more than one outcome source

^f^ Divides studies by quality scores above (“Higher”) or below (“Lower”) the median score of 10

^g^ “Yes” if the study only included participants who were previously reported to have some condition related to hyperactivity

^h^ “Yes” if the study only included participants who were previously reported to have had some behavioral improvements on a synthetic food dye elimination diet

^i^ Typically a preservative like benzoic acid

^j^ “Yes” if the average number of dietary infractions was low (e.g., < 2 per week)

The most frequent study locations were in the US (44%), followed by the UK (22%), and Australia and Canada (15% each). The mean number of participants was 44 (range 1–297). All studies used cross-over designs. In the cross-over design, each subject receives each treatment (including placebo) and, thus, the subjects serve as their own controls, which minimizes bias and confounding. Most challenge studies were double-blinded and the cross-over design was randomized, although in two studies the use of blinding was unclear. Randomization was either not done or was unclear in seven studies. Six studies assessed tartrazine only, whereas the rest studied mixtures of common dyes. The average dose assessed was 55.8 mg/day (range 1.2 to 250 mg/day, doses relevant to children’s exposure in the US). In all but one challenge study, participants were placed on an elimination diet during the study. Most studies (70%) used a validated or otherwise commonly accepted metric to assess neurobehavioral outcomes, with the most common being the Conners Parent scale.

Sixteen (64%) out of 25 challenge studies identified some evidence of an association and in 13 (52%), the association was statistically significant (Fig. 1 and Table 2; Table A.2). Associations (either large effect sizes or statistically significant results) were most commonly identified in studies that assessed neurobehavioral outcomes using information from the child’s parents. Out of eight challenge studies that provided results for both parents and teachers, four found associations only when examining parent reports [5–8], one found associations for both parent and teacher reports [9], two did not report an association for any outcome metric [10, 11], and one found an association only for another metric [12].

Positive associations were also more frequently reported in studies published after the year 1990 (83.3 vs. 57.9%, *p* = 0.26), in studies that used validated metrics for assessing outcome (70.6 vs. 50.0%, *p* = 0.17) and in studies with larger numbers of participants (see Fig. 1 and Table 2). The reason why more recent studies tended to report associations compared to earlier studies is unclear.

While two positive studies tested mixes of dyes plus preservatives [13, 14], the large majority did not include preservatives and many of these (59.1% overall), identified associations between these dyes and adverse effects on neurobehavior with 10 of them reporting associations that were statistically significant [5, 7, 15–17].

Rowe and Rowe [17] saw a dose-response pattern between increasing doses of 1, 2, 5, 10, 20, and 50 mg of Yellow No. 5 (tartrazine) per day and worsening behavioral scores. Only two other studies reported information on dose-response, one using multiple dyes and one with Yellow No. 5 alone, with neither finding a clear dose-response pattern [18, 19] However, Rowe and Rowe used many more doses and had a larger sample size than the other two studies. These differences and other study design issues may have affected whether a dose-response could be seen.

We could not divide studies based solely on age as there was a wide range of ages studied with broad overlap across studies reviewed. However, based on sensitivity analyses examining age, in three studies, results varied minimally [11, 17, 20], while in three others, greater effects were seen in younger participants [5, 14, 21].

#### Nigg et al., 2012 meta-analysis

A high-quality meta-analysis [22] is supportive of the hypothesis that synthetic food dye exposures is associated with adverse behavioral effects in children. This study identified statistically significant summary associations for findings based on parent reports or on attention tests, with effect sizes about one-sixth to one-third of those seen for improvements from ADHD medications. Nigg et al. estimated that 8% of children with ADHD may have symptoms related to synthetic food dyes. Our report evaluated the same studies used in the Nigg et al. meta-analysis as well as two pilot or preliminary reports [7, 19], two studies with only 1–2 participants [8, 16], and a study published after the meta-analysis was published [10]. These five studies reported mixed results. It is unlikely their inclusion in a meta-analysis would dramatically affect its results because most of these studies had small sample sizes. Additionally, the Lok et al. study [23] did not present means and standard deviations for analyses comparing placebo to artificial food dyes, and as such would be difficult to include in meta-analysis with most other studies.

#### Bias and confounding

As documented in Tables S.2-S.4 we performed extensive evaluations of quality for each study. One strength of our findings is that they are based on clinical trials with cross-over designs and placebo control. Non-compliance can lead to exposure misclassification in clinical trials, but we found that infraction rates were generally low in the studies when they were reported. Potential confounding can be markedly reduced with the use of cross-over designs since subjects are being compared to themselves. Bias that may be introduced by the expectations of the researchers and participants is minimized by use of blinding and placebo control. We performed a sensitivity analysis in which we only included studies that were double-blinded and had the cross-over randomized, and found that our conclusions were similar to that of our analysis that included all studies (Table 2, rows for RCDP).

Recruitment strategies and participation rates were not always clearly described in the studies, and most seemed to involve convenience samples. The use of convenience samples or low participation rates can introduce bias. However, in studies in which the participants, parents, and others were blinded, we found no clear evidence or obvious reason that convenience sampling or low participation might cause false positive results. While convenience sampling and low participation rates might affect the generalizability of some studies, we see no reason why they would affect the ability of a study to examine whether at least some children might be adversely affected by synthetic food dyes, especially given the cross-over design.

Adjustments for publication bias by Nigg et al. [22] attenuated summary effect sizes in the meta-analysis, although several remained statistically significant. However, these adjustment methods are imperfect. In addition, given the widespread interest in the potential health effects of synthetic food dyes, it seems unlikely that well-conducted clinical trials would remain unpublished resulting in publication bias.

#### Susceptibility

From the studies reviewed, it appears that not all children react to the dyes with adverse behavioral outcomes. Possible explanations for this sensitivity are not clear. Studies that included only children who were previously diagnosed with hyperactivity were not more likely to report positive associations between synthetic food dye exposure and poorer behavioral outcomes. Stevenson et al. [24] found that children (both 3 year-olds and 8/9 year-olds) with certain polymorphisms in histamine degradation genes had greater adverse responses to synthetic food dyes. In addition, gene polymorphisms in the dopamine transporter gene in 8/9 year-old children moderated the effects of the food dyes. Since histamine plays a role as a neurotransmitter in the brain and is involved in wakefulness, polymorphisms in the histamine degradation genes are a plausible basis for varied behavioral sensitivity to dyes associated with histamine release. Replication of this study and further research of the impacts of gene polymorphisms on response to food dyes are needed.

### Review of animal toxicology studies

Animal toxicology studies were used by FDA as the basis for regulatory risk assessments of food dyes [25]. All current dye registrations were made between 1969 and 1986 based on studies performed 35 to 50 years ago. These studies were not designed to assess neurobehavioral endpoints. Dye registration was accompanied by derivation of an “acceptable daily intake” (ADI) based on these studies. FDA ADIs have not been updated since original dye registration, although there have been several reviews of specific effects since then, the latest in 2011 [25].

**Fig. 2:**
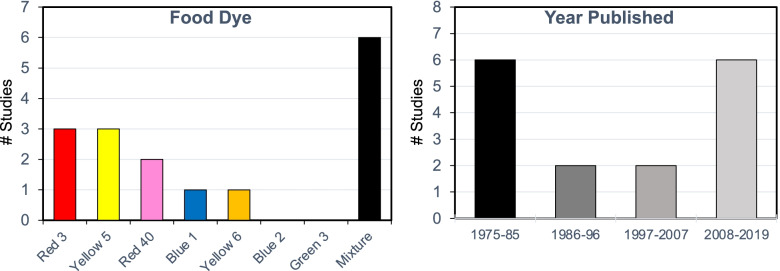
Number of animal developmental neurobehavioral toxicity studies by dye and year

**Fig. 3:**
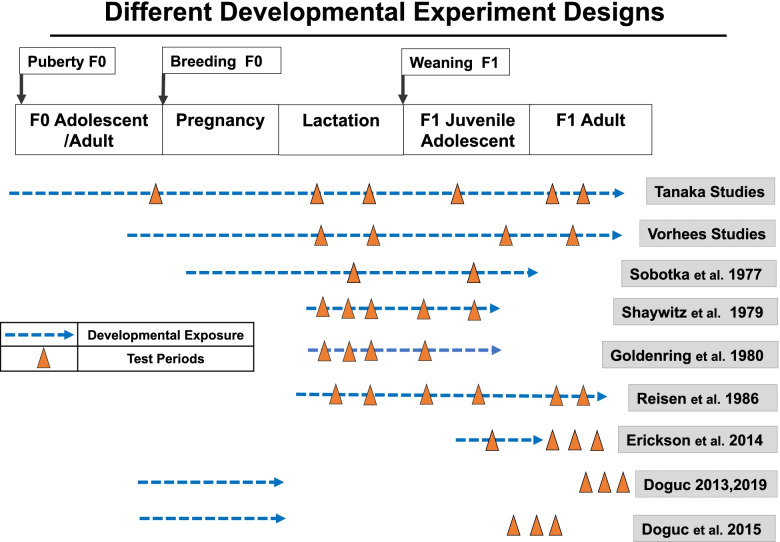
Experimental designs of developmental neurotoxicity studies in animals with synthetic food dye exposures

Our review of animal toxicology studies was intended to examine neurobehavioral toxicity of food dyes and included any study administering one or more of the FDA registered food dyes and measuring a behavioral endpoint. We obtained 25 reports from the peer-reviewed literature. Two reports could not be reviewed due to lack of study information. The 23 studies reviewed had the following characteristics:

- Rodent models (rats or mice)
- Oral administrations (diet or gavage)
- Dosing with individual dyes (14 studies) or dye mixtures (9 studies) (Fig. 2)
- Dosing included at or below that in studies used to establish FDA ADIs
- Durations ranging from a single dose to lifetime daily dosing (Fig. 3)
- Behavioral endpoints including preweaning motor development, spontaneous motor activity and/or learning and memory tests
- Comparison of dosed and control groups

The study designs varied (Fig. 3) and included exposures during prenatal, infant, juvenile and adult life stages, and examined neurobehavioral effects during development and/or adulthood. Due to the wide range of designs, an overall integration of findings was not possible but a broader picture of the potential for food dye neurobehavioral toxicity is seen. Details of the studies are presented in Table A.5 and A.6. Detailed evaluation and interpretation of each study is reported in the OEHHA document [1]. First author and dates of publication are shown.

Details from all the studies reviewed in this section are shown in Table A.5.

Findings from these studies have greatly advanced our knowledge of neurobehavioral effects of synthetic food dyes:

- Long term consequences of exposure during pregnancy [26–29]. This is the first research using the classical developmental neurotoxicology (DNT) design where exposure begins during pregnancy to identify long-term effects of perinatal exposure. Prior regulatory developmental toxicology studies have been limited to effects on mortality, malformation, and growth. There are no studies in humans using exposure in pregnancy.
- Effects of synthetic food dyes on behavior in adult rats after a single administration [30, 31]. These are the only available animal studies measuring behavior shortly after a single dye administration.
- Behavioral effects when synthetic food dyes are administered at juvenile/adolescent life stages [32, 33]
- Effects on behavior in adult rodents with chronic exposures [31, 34–36]. Due to the emphasis on behavioral effects in children, more general studies of neurobehavioral toxicity in adults have been lacking but have recently been undertaken in animal models.
- Prevention of effects of synthetic food dyes on behavior by antioxidants [35, 36]. This line of investigation has also been pursued for other aspects of dye toxicity [37–45]
- Brain changes associated with behavioral effects [26, 29–31, 34–36]. Emerging research in the last 10 years has begun to explore effects of synthetic food dye exposures on the brain at doses that affect behavior.

#### Individual dye studies

A series of neurobehavioral studies of individual dyes has been performed by one laboratory in Japan for 5 of the 7 food dyes approved for use in the US [46–51]. These studies used lifetime exposure beginning prior to parental mating. Details of the studies can be found in Table A.5. For three of the dyes, behavioral effects were identified at doses below those producing the toxicological effects used to establish the FDA ADIs. Several considerations limit the use of these studies in assessing food dye risk to children, including reproductive toxicity in the studies, multiple life stage exposure, dosing both before and during testing, and lack of litter-based statistics for preweaning endpoints.

Eight studies were from US laboratories, all published prior to 1987. We did not find any programmatic investigator-initiated research on neurobehavioral effects of food dyes currently being performed in the US.

The US FDA supported early studies of three synthetic food dyes (Yellow No. 5, Red No. 3, Red No. 40) that also used lifetime exposures beginning prior to or shortly after conception and continuing through adult animal testing [52–54]. Dosing was based on known non-behavioral toxicity of the dyes. Behavioral effects were reported for Red No. 3 [54] and Red No. 40 [53] using an extensive test battery.

The other 5 studies of individual dyes were conducted more recently and administered individual dyes to postpubertal (adolescent and adult) rodents [30, 31, 34–36]. These investigators were interested in specific hypotheses about food dye mechanism of action and included brain assays: brain microhistomorphometry [35, 36]; measures of oxidative stress [34]; and measures of influence on the serotonin system [30, 31].

#### Mixture studies

Early studies used dye mixtures designed to parallel US food dye exposure at that time [33, 55–58]. More recent studies used dosing based on a multiple of regulatory ADIs [27–29, 32]. Study details are presented in Table A.6. Animal mixture studies, like children’s mixture studies, are valuable for hazard identification, but not for ADI development, which is based on each dye individually.

The two studies using synthetic food dye mixtures that are most relevant to the studies in children reported behavioral effect during dye administration to immature rats [32, 33]. Both studies reported effects on regulation of spontaneous motor activity. Shaywitz et al. [33] used a mixture based on human exposure and found greater activity in rats dosed at twice the estimated average exposure at that time in children. Erickson et al. [32] found increased movement time using a mixture of dyes in drinking water, each dye at a dose less than 2 times the FDA ADI.

One series of studies examined exposure to a mixture of synthetic food dyes during pregnancy [27–29]. The 9 dyes were administered at either the JECFA ADI [28] or 100 times the JECFA ADI [27, 29]. Six of the seven FDA registered food dyes were included. Effects on activity and emotionality were reported with testing of one-month old (early adolescence) and three-month old (adulthood) offspring, but learning and memory tests were not affected.

#### Behavioral endpoints

Behavioral assessments were primarily conducted in a few domains: preweaning motor development (4 studies), spontaneous motor activity (21 studies), and trial-based learning and memory tests (18 studies). Some recent studies included emotionality tests [27–29, 32].

Spontaneous motor activity, a sensitive and widely used test in developmental neurotoxicology, was the most frequently used test in animal dye studies because of the findings of hyperactivity in children’s studies. While the test apparatus and specific endpoints affected (vertical/horizontal activity, speed, distance, duration) varied, altered regulation of activity was seen in 17 of the 21 of the studies.

Sensitivity of learning and memory tests in developmental neurotoxicology is less consistent [59, 60]. For the food dye studies we examined, tests included shock-motivated avoidance, food motivated mazes, and water mazes, with 12 of 18 studies reporting dye effects. Our review found that many of the test results could not be used for risk assessment due to design and statistical issues. For example, some studies did not use litter-based statistics.

#### Brain assays in behavioral studies

Many animal studies we reviewed conducted brain assays that evaluated a number of parameters with a focus on neurotransmitter systems. Early studies did not identify effects of synthetic food dye exposures on tissue catecholamine neurotransmitter concentrations [33, 55–57]. More recent studies identified effects on gene receptor expression, enzyme activity in neurotransmitter systems, and localized changes in neurotransmitter levels [26, 29–31]. Also, brain histomorphology assessed with contemporary methods has identified effects (decreased medial prefrontal cortex volume, decreased numbers of glia and neurons, changes in dendritic morphology) of the two most used food dyes, Red No. 40 and Yellow No. 5 [35, 36]. Protective effects of antioxidants [35, 36], as well as changes in brain anti-oxidant defense systems [34] provide evidence for oxidative stress as a mechanism of toxicity. Two other papers with no behavioral measures found markers of oxidative stress in the brain after in vivo treatment of rats with Yellow No. 5 [61, 62].

Data from a human study provide evidence for a mechanism involving the neurotransmitter histamine. The investigators demonstrated that polymorphisms in the histamine degradation gene for histamine-N-methyltransferase influences response to a dye mixture [24]. In addition to its role in the inflammatory process, histamine is recognized for its role in regulating synaptic transmission alone and in concert with other neurotransmitters [2].

Considering both in vivo and in vitro research, other potential pathways for food dye neurotoxicity have been suggested [63–65].

- Endocrine (thyroid, estrogen) mediated effects
- Interference with neuronal proliferation and differentiation
- Effects secondary to general physiological toxicity
- Immune mediated effects
- Interference with nutrient bioavailability

The relevance of the animal toxicology findings to humans ingesting synthetic food dyes in food and medications would be better understood with more information about food dye toxicokinetics. In particular, the breakdown of azo dyes in the gut prior to absorption requires toxicological examination of metabolites. Future studies should evaluate whether the parent compounds act on the gut to influence behavior via the gut-brain axis [66].

### Hazard characterization

The studies that form the basis of the FDA (and JECFA) ADIs are many decades old and as such were not capable of detecting the types of neurobehavioral outcomes measured in later animal studies, or in clinical trials in children consuming synthetic food dyes.

Nonetheless, OEHHA first compared the US FDA ADIs and the No-Observed-Adverse-Effect Levels (NOAELs) from which they were derived to NOAELs from the animal toxicology studies that were reviewed [1]. Next, we compared the estimated food dye exposures (mg/kg/d) from food consumption to available regulatory benchmarks in a traditional Hazard Index approach for noncancer health effects. The Hazard Index approach divides estimated exposures by a toxicity benchmark. If that ratio is greater than 1, then it is indicative of a possible risk of adverse noncancer effects. Finally, we compared the ADIs to NOAELs and Lowest-Observed-Adverse-Effect Levels (LOAELs) observed in the few key animal and human studies of sufficient quality. This comparison should help inform future revisions of the ADIs aimed at protecting children from neurobehavioral effects.

#### Comparing neurobehavioral effect levels to FDA ADI NOAELs

To derive the ADI for each dye, US FDA divided NOAELs reported by investigators from animal studies by a factor of 100. While reviewing animal neurobehavioral toxicology studies, we compared the effective doses (LOAELs) to animal NOAELs used by US FDA to derive human ADIs (hereinafter referred to as ADI NOAELs). The purpose of this comparison was to see if neurobehavioral effects were found at doses that FDA determined were not causing effects in the older general toxicology studies**.** Tables 3 and 4 presents these comparisons for both developmental and adult neurotoxicology studies where a single dye was administered.

**Table 3:** Comparison of US FDA ADI and effective oral doses from developmental studies with individual dyes

	Vorhees et al., 1983a	Tanaka 2001	Vorhees et al.,1983b	Tanaka 1994	Tanaka et al., 2012	Sobotka et al., 1977	Tanaka et al., 2006	Tanaka et al., 2008	Tanaka 1996
**Dye**	Red No. 3	Red No. 3	Red No. 40	Red No. 40	Blue No. 1	Yellow No. 5	Yellow No. 5	Yellow No. 5	Yellow No. 6
**FDA ADI**	2.5	2.5	7.0	7.0	12.0	5.0	5.0	5.0	3.75
**100 X ADI**^**a**^ **(mg/kg/d)**	250	250	700	700	1200	500	500	500	375
**Study Doses (as % diet) NOAEL**^**b**^ **LOAEL**^**c**^	0, 0.25^**c**^, 0.5, 1.0	0, 0.005**,** 0.015^**b**^, 0.045^**c**^	0, 2.5^**c**^, 5.0, 10.0	0, 0.42, 0.84, 1.68^**b**^	0, 0.08^**c**^, 0.24, 0.72	0, 1.0, 2.0^**b**^	0, 0.05, 0.15, 0.45^**b**^	0, 0.05, 0.15, 0.45	0 0.15, 0.30, 0.60^**b**^
**Study** ^**d**^ **NOAEL or LOAEL in mg/kg/d**	LOAEL 125^**e**^	NOAEL 24	LOAEL 1250^**e**^	NOAEL 3534	LOAEL 127	NOAEL 1000^**e**^	NOAEL 841	Significant trend tests only	NOAEL 1146
***LOAEL < FDA ADI NOAEL***	***yes***	***yes***	***no***	***no***	***yes***	***no***	***no***	***N/A***	***no***

Effective doses are those at which statistically significant differences between dose group and control group were reported by authors**.** Endpoints are behavior or brain measures

^**a**^NOAEL used to derive FDA ADI

^**b**^NOAEL for study

^**c**^LOAEL for study

^**d**^For studies from the Tokyo Metropolitan Laboratory of Public Health, for NOAELS without LOAELS, the mean value for males and females were used. For LOAELs and NOAELs with LOAELs, the value for the sex affected at the LOAEL was used

^**e**^ Calculated by OEHHA using standard assumptions about food intake and body weight

**Table 4:** Comparison of US FDA ADI and effective oral dose from adult studies with individual dyes

	Tanaka 2001	Dalal and Poddar 2009	Dalal and Poddar 2010	Noorafshan et al., 2018	Tanaka et al., 2012	Tanaka et al., 2006	Tanaka et al., 2008	Gao et al., 2011 (rats)	Gao et al., 2011 (mice)	Rafati et al., 2017	Tanaka 1996
**Dye**	Red No. 3	Red No. 3	Red No. 3	Red No. 40	Blue No. 1	Yellow No. 5	Yellow No. 5	Yellow No. 5	Yellow No. 5	Yellow No. 5	Yellow No. 6
**FDA ADI**	2.5	2.5	2.5	7.0	12	5.0	5.0	5.0	5.0	5.0	3.75
**100 x ADI**^**a**^ **mg/kg/d**	250	250	250	700	1200	500	500	500	500	500	375
**Study Doses** **NOAEL**^**b**^ **LOAEL**^**c**^	0, 0.005, 0.015 ^**b**^, 0.045^**c**^ % diet	0, 1^**b**^, 10^**c**^, 100, 200 mg/kg/d	0, 1^**b**^, 10^**c**^, 100, mg/kg/d	0, 7^**c**^, 70 mg/kg/d	0, 0.08^**c**^, 0.24, 0.72% diet	0, 0.05^**b**^, 0.15^**c**^, 0.45% diet	0, 0.05, 0.15, 0.45^**b**^ **%** diet	0, 175^**b**^ 350 700 mg/kg/d	0, 125^**b**^ 250 500 mg/kg/d	0, 5^**c**^, 50 mg/kg/d	0, 0.15, 0.30, 0.60 ^**b**^ % diet
**Study**^**d**^ **NOAEL or LOAEL in mg/kg/d**	NOAEL 28^**e**^	NOAEL 1.0	NOAEL 1	LOAEL 7.0	LOAEL 122^**e**^	NOAEL 73^**e**^	NOAEL 824^**e**^	NOAEL 175	NOAEL 125	LOAEL 5 & 50	NOAEL 1052^**e**^
***LOAEL < FDA ADI NOAEL***	***yes***	***yes***	***yes***	***yes***	***yes***	***yes***	***no***	***yes***	***yes***	***yes***	***no***

Effective doses are those at which statistically significant differences between dose group and control group were reported by authors. Endpoints are behavior or brain measures

^**a**^NOAEL used to derive FDA ADI

^**b**^NOAEL for study

^**c**^LOAEL for study

^**d**^For studies from the Tokyo Metropolitan Laboratory of Public Health (Tanaka studies), for NOAELS without LOAELS, the mean value for males and females were used. For LOAELs and NOAELs with LOAELs, the value for the sex affected at the LOAEL was used

^**e**^For studies using % diet as dosing metric, doses in mg/kg/d were calculated by OEHHA from data on food consumption and body weight provided in the paper

#### Comparing food dye exposures to available regulatory benchmarks

OEHHA [1] derived exposure estimates based on NHANES 2015–2016 Dietary Interview data, and information on food dye concentration data sourced from Doell et al. [67]. We calculated single-day and two-day average cumulative daily synthetic food dye intake estimates (mg/person/day) for the following demographic categories:

- Pregnant women 18 years and older
- Women of childbearing age (18–49 years)
- Children: 0- < 2 years, 2- < 5 years, 5- < 9 years, 9- < 16 years, and 16–18 years

We estimated daily synthetic food dye intakes (mg/person/day) for

- **The typical-exposure scenario**, which represents exposure to a given FD&C batch-certified synthetic food dye for a typical consumer, an individual who may not always eat products with the lowest or highest levels of that food dye but some combination of both.
- **The high-exposure scenario**, which represents the highest exposure where the individual is only consuming products with the highest levels of that food dye.

We divided each individual’s FD&C batch-certified synthetic food dye intake estimate (mg/person/day) by their body weight (kg) reported in NHANES 2015–16 [68] to produce synthetic food dye dose estimates in units of mg/kg/day. The most commonly consumed dyes for the various age ranges of children expressed as the mean of typical-exposure scenario estimates were Red No. 40 (ranged from 0.11 to 0.3 mg/kg-day), Red No. 3 (ranged 0.02 to 0.54 mg/kg-d), Yellow No. 5 (ranged from 0.05 to 0.19 mg/kg-d) and Yellow No. 6. (ranged from 0.05–0.20 mg/kg-d) [1]. The 95th percentile of the high-exposure scenario estimates ranged from about 1 to 8 mg/kg-day for these four dyes. Children’s exposures tended to be higher than adult women.

We compared the synthetic food dye dose estimates to the US FDA and JECFA ADIs (Table 5) by calculating the ratio of the dose estimates to the established ADIs [25, 69–72] as the Hazard Index. Hazard index > 1 signifies that the food dye exposure estimates (mg/kg/day) exceeded the established ADI.

**Table 5:** ADIs in mg/kg/day from US FDA and JECFA

	US FDA	JECFA (WHO)^**a**^
Yellow 5	5.0	0–10
Yellow 6	3.75	0–4
Red 3	2.5	0–.1
Red 40	7.0	0–7
Blue 1	12.0	0–6
Blue 2	2.5	0–5
Green 3	2.5	0–25

^**a**^ JECFA presents their ADIs as a range from 0 to a positive value

With the exception of FD&C Red No. 3, all exposure estimates (mg/kg/day) from foods were below the US FDA or JECFA ADIs. The Hazard Indices (HI) that exceeded 1 for Red No. 3 are bolded in Table 6. Children’s single day mean FD&C Red No. 3 exposure estimates for typical- and high-exposure scenarios ranged from 0.01 to 0.60, not exceeding the FDA ADI of 2.5 mg/kg-day. The 95th percentile exposure estimates ranged up to 3.16 (although it represents few children). For several age categories the mean single day typical- and high-exposure scenarios exceeded the JECFA ADI of 0.1 mg/kg-day, with HI ranging from 0.21 to 15; the 0 < 2 year age category had the highest HI.

**Table 6:** Ratios of the FD&C Red No. 3 intake compared with US FDA and JECFA ADIs

FD&C Red No. 3	Typical-exposure scenario				High-exposure scenario			
	FDA Ratio Mean	FDA Ratio 95th%	JECFA Ratio Mean	JECFA Ratio 95th%	FDA Ratio Mean	FDA Ratio 95th%	JECFA Ratio Mean	JECFA Ratio 95th%
Pregnant women								
Day 1	0.01	0.09	0.29	**2.28**	0.02	0.27	0.60	**6.66**
Day 2	0.008	0.02	0.20	0.41	0.01	0.02	0.23	0.54
2 -Day average	0.008	0.05	0.20	**1.14**	0.01	0.13	0.35	**3.33**
Women 18–49 years								
Day 1	0.01	0.03	0.27	0.78	0.02	0.03	0.38	0.81
Day 2	0.01	0.04	0.29	**1.02**	0.02	0.04	0.38	**1.02**
2 -Day average	0.01	0.03	0.18	0.72	0.01	0.03	0.24	0.80
Children (0- < 2 years)								
Day 1	0.01	0.04	0.28	0.90	0.01	0.04	0.32	**1.11**
Day 2	0.21	**1.93**	**5.35**	**48.3**	0.60	**3.16**	**15.0**	**79.0**
2-Day average	0.07	0.03	**1.73**	0.68	0.19	0.03	**4.72**	0.68
Children (2- < 5 years)								
Day 1	0.08	0.07	**1.89**	**1.85**	0.19	0.08	**4.85**	**1.90**
Day 2	0.02	0.06	0.56	**1.56**	0.03	0.07	0.84	**1.68**
2-Day average	0.03	0.04	0.70	0.90	0.07	0.04	**1.66**	0.90
Children (5- < 9 years)								
Day 1	0.03	0.04	0.64	**1.12**	0.04	0.06	**1.05**	**1.38**
Day 2	0.04	0.08	**1.09**	**1.98**	0.07	0.09	**1.72**	**2.14**
2-Day average	0.02	0.05	0.62	**1.22**	0.04	0.09	0.98	**2.28**
Children (9- < 16 years)								
Day 1	0.03	0.06	0.87	**1.61**	0.08	0.13	**1.96**	**3.19**
Day 2	0.03	0.06	0.87	**1.38**	0.06	0.06	**1.52**	**1.44**
2-Day average	0.02	0.06	0.55	**1.60**	0.04	0.17	**1.05**	**4.21**
Youth (16–18 years)								
Day 1	0.02	0.09	0.49	**2.14**	0.03	0.09	0.69	**2.14**
Day 2	0.007	0.02	0.17	0.57	0.01	0.03	0.21	0.80
2-Day average	0.007	0.02	0.18	0.54	0.01	0.02	0.24	0.62

US Food and Drug Administration (US FDA ADI = 2.5 mg/kg/day)

*JECFA* Joint FAO/WHO Expert Committee on Food Additives (JECFA ADI = 0.1 mg/kg/day)

#### Comparing US FDA ADIs to key neurobehavioral studies

There are several animal studies and one human study that could be used to evaluate whether existing ADIs are protective of neurobehavioral effects for Red No. 3, Red No. 40, Yellow No. 5 and Yellow No. 6. No suitable studies of green or blue dyes were found for this comparison.

##### Red no. 3

Tanaka et al. [48] conducted a developmental toxicity study of Red No. 3 where various doses were administered via diet from preconception through PND 63 and reported increased activity measurements in female offspring. For adult female dams, more turning was reported in the high-dose group than in controls. Activity in male offspring was affected at 3 weeks of age (*p* < 0.01 for linear dose trend), but not at 8 weeks of age. In the female offspring at 8 weeks, statistically significant dose-dependent dye-induced increases in activity were seen, but not at 3 weeks of age. These included number of activity bouts, distance traveled in each bout, greater speed, total time moving and total distance. This interesting finding of greater activity is particularly valuable because of the absence of more severe developmental toxicity.

The NOAEL was 24 mg/kg/day for the female offspring. This NOAEL is a factor of 10 higher than the FDA ADI of 2.5 mg/kg/day. If one were to apply the same methodology as US FDA (dividing the NOAEL by a factor of 100) to derive an ADI, the resulting ADI would be a factor of 10 lower.

The studies by Dalal and Poddar [30, 31] (Table A.5) provide unique information on brain serotonin pathway changes, and on behavioral changes in young adult animals either following single gavage administration or following 15 or 30 day exposures to Red No. 3. In their first study, the investigators measured activity (vertical rearing frequency detected automatically) for 5 min at 30 to 60 min intervals up to 9 h post-dosing after single gavage doses of 0, 1, 10, 100 or 200 mg/kg. A dose-dependent pattern of diminished activity was observed that reached a low at 2 h after dye administration and then returned to baseline by 7 h (Fig. 1 in Dalal and Poddar (2009)). The effect of diminished activity was replicated in an experiment demonstrating reversal of this effect by inhibitors of monoamine oxidase (MAO), the enzyme that metabolizes serotonin. In the second report, the investigators administered the same doses daily for a period of 15 or 30 days and activity was measured following the last administration. Following the 15 or 30 day treatments, activity was increased rather than decreased in a dose-dependent fashion (Fig. 1 in Dalal and Poddar (2010)). One explanation for these contrasting results is the role of two neuronal corticotrophin releasing factor (CRF) receptors that determine an active versus passive response to stress [73]. The NOAEL from these studies is 1 mg/kg/day based on changes in vertical activity in male rats, on increased serotonin levels in specific brain regions, and increased plasma cortisone levels. The NOAEL of 1 mg/kg/day in these studies is lower than the FDA ADI of 2.5 mg/kg/day. If one were to use a 100-fold safety factor with this NOAEL, the ADI would be 0.01 mg/kg/day.

##### Red no. 40 and yellow no. 5

Noorafshan et al. [35] administered Red No. 40 to adult male rats (*N* = 10 per dose group) at doses of 0, 7, or 70 mg/kg/day (Table A.6) with and without 200 mg/kg/day of the anti-inflammatory molecule taurine, by gavage for 6 weeks. Both Red No. 40 treated groups performed more reference memory errors and working memory errors in the radial arm maze than controls (*p* < 0.01). Taurine administration mitigated this effect. Histomorphology and stereology found that, in the high dose Red No. 40 group, the medial prefrontal cortex volume was smaller, and there were fewer neurons and glial cells in this brain area. Interpretation of these results is somewhat complicated by the lack of information on body weight and brain weight. The LOAEL is 7 mg/kg/day for this study, which is the same as the US FDA and JECFA ADI of 7 mg/kg/day.

These investigators used the same protocol to evaluate the effect of another azo dye, Yellow No. 5 [36]. Adult male rats (*N =* 10 per dose group) were gavaged with Yellow No. 5 at 0, 5, or 50 mg/kg/day for 7 weeks with and without vitamin E. Exploration time in the novel object test was decreased at the high dose (p < 0.01). More days were required for Yellow No. 5 treated rats (low- and high-dose groups were combined) to reach the learning criterion in the radial arm maze test, and more errors occurred during the learning and retention phases. The brain assays demonstrated a smaller volume of the medial prefrontal cortex in the high-dose group, and lower cell count and shorter dendrites with lower spine density at both doses; qualitative alterations in cell shape were described. These effects were ameliorated by concomitant administration of the antioxidant vitamin E. The LOAEL was 5 mg/kg/day, based on morphometry, the same as the US FDA ADI of 5 mg/kg/day and lower than the JECFA ADI of 10 mg/kg/day. If this study were to be used as the basis for setting an ADI, the resulting ADI would be considerably lower than the existing ADI. Changes in the medial prefrontal cortex can be directly related to the cognitive performance of the animals, as this part of the rodent brain is involved in spatial memory, decision-making and attention [35, 74], and may predict similar effects in children.

One study in children used several doses and demonstrated a dose response effect on behavioral scores for Yellow No. 5 [17]. For this study, the investigators recruited 34 children whose parents had brought them to the Royal Children’s Hospital in Melbourne to be evaluated for hyperactivity and 20 children whose parents had no concern about behavior. The children, ranging in age from 2 to 14 years, were enrolled in a double blind, placebo-controlled repeated measures study of the effects of Yellow No. 5 on behavioral score. The investigators developed a Behavioral Rating Inventory for this study that included 11 items measuring irritability, 9 items that measured sleep disturbance, 4 items that measured restlessness, 3 items that measured aggression and 3 items that measured attention span. In addition, the investigators also used the Conners 10-item Abbreviated Parent-Teacher Questionnaire to assess behavior, which focuses on attention related problems. Children were placed on a dye-free diet for at least 6 weeks before the trial, and then given doses (randomly) of 0, 1, 2, 5, 10, or 20 mg Yellow No. 5 with 2 days in between each dosing. Parents rated the behavior daily using the two instruments.

The investigators found 24 children who had significant behavioral responses to dye challenge, based on ranking the behavioral scores for the six dye-challenge days paired with a set of placebo days; these children were labelled as reactors. The mean behavioral scores on dye-challenge days were significantly different than the scores for the placebo (day before) challenge for all dose/placebo pairs (*p* < 0.05) in the reactors, while the nonreactors showed random fluctuations in behavioral scores. Using repeated measures ANOVA on the six dye-challenge scores with reactors and nonreactors as the between-groups factor, the authors report a significant between-groups effect (*p* < 0.001). There was a dose-dependent effect and the mean score difference between the reactor and the nonreactor groups were significant at doses of 2 mg and higher (p < 0.05). There were no significant differences in mean behavioral rating between the groups on the placebo days. OEHHA identifies 1 mg tartrazine as a NOAEL. The children ranged from 2 to 14 years, with a mean of 7 years. To determine a NOAEL dosage, OEHHA divided the NOAEL of 1 mg by a reference body weight of 25.5 kg for the mean age of 7 years (US EPA, 2011, Table 8–10, based on NHANES 1988–1994); a NOAEL dosage of 0.04 mg/kg/day is obtained. This NOAEL is more than 100-fold lower than the US FDA ADI for Yellow No. 5 of 5 mg/kg/day.

While not all of the human trials demonstrated effects of mixtures of food dyes or of Yellow No. 5 on behavior, the findings of Rowe and Rowe [17] are supported by some of the other clinical trials in children (Table 7). Note that in all these studies, effects were observed at estimated doses lower than the US FDA ADI for Yellow No. 5 of 5 mg/kg/day. One study [9] reports that in a six-week open trial of the Feingold diet in 55 subjects, ages 3 to 15 years, who had been suspected of reacting to food dyes, 40 children demonstrated improvement when on the Feingold diet, based on assessment of attention span, activity level, distractability, frustration tolerance, and social and manipulative skills by therapists, and teacher and parent questionnaires. In the same study, 8 of the children were challenged with Yellow No. 5 using a double-blinded cross-over design, and two of these children were observed to exhibit strong behavioral responses to the dye. Based on reference body weights for children ages 3 to 15 years, the dosages employed in that study [9] would have been 0.9–2.7 mg/kg/day. In a double-blind crossover study of 22 children, 4 to 8 years of age, both objective tests for attention and parent and teacher ratings (Conners Parent Teacher Rating Scale) were administered before and after a 4 week dye-free diet, after a 2 week Yellow No. 5 (5 mg daily) challenge and after a 4 week washout dye-free diet [7]. The investigators report statistically significant effects of Yellow No. 5 based on parental ratings in a subgroup of children whose mothers had reported improved behavior while on the elimination diet. The dose for this range of ages and body weights to the children would be 0.2 to 0.3 mg/kg/day. Levy and Hobbs [75] reported that mothers’ ratings using the Conners scale were an average of 13% lower when children (*N* = 8) ate placebo cookies compared to those containing Yellow No. 5, in a 2 week crossover trial with daily ratings by parents for a 3 h period after eating the cookies. While there were no statistically significant differences noted, the authors reported that this effect “just failed to reach the .05 level of significance”. The dose of Yellow No. 5 in this study was about 0.1 to 0.2 mg/kg/day.

**Table 7:** Doses of Yellow No. 5 that elicited effects in children’s clinical trials

Study	Rowe and Rowe (1994)	Rowe (1988)	Levy et al. 1978	Levy and Hobbs (1978)
**Administered amount**	0, 1, 2, 5, 10, or 20 mg	50 mg	5 mg	4 mg
**Estimated effective dose (mg/kg/day)**	0.04^a^	0.9–2.7^b^	0.2–0.3 ^b^	0.1–0.2 ^b^

^a^ LOAEL dose estimated for the mean age of 7 years

^b^ single dose studies, dose estimated for reported range of ages of children

Taken together, these studies provide support for an effect of Yellow No. 5 on behavior and for use of a neurobehavioral endpoint to determine a safe level of exposure for Yellow No. 5 to protect children who respond to this food dye.

##### Yellow no. 6

There is only one study of Yellow No. 6 with neurobehavioral endpoints [47]. Some neurobehavioral effects in offspring were reported for preweaning development and maze learning, but it was not possible to draw firm conclusions due to the statistical approach and varying group sizes in the study.

Goldenring et al. demonstrated that sulfanilic acid (1 mg/kg/day I.p.), a common metabolite of the azo food dyes Yellow No. 5 and Yellow No. 6, increased activity in pups following direct administration assessed three times during a treatment extending throughout juvenile development [55].

Honohan et al. reported gastrointestinal absorption of sulfanilic acid of 37.4% [76, 77]. The 1 mg/kg intraperitoneal dose of sulfanilic acid used by Goldenring et al. would be equivalent to 2.7 mg/kg produced in the gastrointestinal tract, which in turn would result from metabolism of 7 mg/kg of orally administered Yellow No. 5. Thus, one could view 7 mg/kg−/day of Yellow No. 6 to be a free-standing LOAEL. This LOAEL is about twice the FDA (3.75 mg/kg/day) and JECFA (4 mg/kg/day) ADIs for Yellow No. 6. The study by Goldenring et al. [55] indicates the ADIs for Yellow No. 6 may not be adequately protective of neurobehavioral effects.

## Discussion

Current evidence from studies in humans, largely from controlled exposure studies in children, supports a relationship between food dye exposure and adverse behavioral outcomes in children, both with and without pre-existing behavioral disorders. There appears to be considerable interindividual variability in the sensitivity to synthetic food dyes. While there were a range of results in the studies we identified, the majority reported at least some evidence of an association, including higher quality studies. Importantly, none of the factors we examined (e.g., parent vs teacher report, publication year, validated outcome metric) explained the majority of the heterogeneity seen across the study results. For example, although a large fraction of the studies published since 1990 reported statistically significant results (5 of 6 challenge studies), many studies published before 1990 also reported statistically significant results (8 of 19). And, while studies using a validated outcome metric were more likely to report associations, several studies without validated outcome metrics reported similar associations. Despite the various study limitations, we were unable to identify strong evidence for any apparent biases or other factors that invalidated the positive results reported in the literature.

Studies of Yellow No. 5 alone provide evidence that this dye affects children’s behavior. Most of the challenge studies involved administering multiple dyes at the same time so no single offending agent could be identified from those studies. Regardless, studies involving mixtures more closely represent real-life scenarios, where most children are exposed to multiple dyes in a single day.

Importantly, impacts on behavior and/or neurotransmitter systems or cellular architecture in the brain have been observed in animal studies. Several studies examining exposures during development, during pregnancy only, or as adolescents or adults reported changes in activity using a variety of metrics either in the offspring or in the adolescent or adult animals. In utero exposure was observed to have behavioral effects in the adult offspring. Thus, the animal literature provides support for behavioral effects of synthetic food dyes, including those most often consumed.

Taken together, the scientific literature supports an effect of synthetic food dye exposures on neurobehavior in children at environmentally relevant exposure levels.

Comparing estimated exposures we derived from the 2015–16 NHANES dietary interview to the FDA and JECFA ADIs revealed that for most dyes we analyzed, exposures do not exceed the ADIs. The exception is Red No. 3, where the Hazard Index based on the mean ranged up to 15 for the youngest age groups (Table 6).

Comparisons of the effective doses in some of the animal studies that measured behavioral or brain effects following exposure to synthetic food dyes indicates that the basis of the FDA ADIs are not adequate to protect neurobehavior in susceptible children. Three of the studies using developmental exposures reported LOAELS that were below the NOAEL that was used for the FDA ADI. Almost all studies in mature animals that measured behavioral changes and/or changes in the brain found effects of the synthetic food dyes at doses lower than the NOAELs used by the US FDA for the derivation of the ADIs. Several studies observe effects on behavior in animals at doses close to or even lower than the existing FDA ADIs. As noted above, the animal studies that form the basis of the FDA ADIs were not capable of detecting the types of neurobehavioral outcomes observed in many human challenge studies.

For four of the dyes with adequate animal studies explicitly reporting neurobehavioral effects, applying results from these studies would result in lower ADIs and likely exceedances of those ADIs from typical food consumption by children. Consumption of over-the-counter medications and vitamins adds to the exposure from foods [78, 79].

If the ADI for Yellow No. 5 were based on the one study that evaluated a dose-response in children for behavioral effects, the ADI would be considerably lower. The human challenge studies provide support for an effect of Yellow No. 5 on behavior and for use of a neurobehavioral endpoint to determine a safe level of exposure for Yellow No. 5 to protect children who respond to this food dye.

It is not possible to compare the results of the animal or human mixtures studies to an ADI for a single dye. However, Erikson et al. [32] reported increased activity in male rats administered synthetic food dye mixtures where each dye was given at less than twice the ADI NOAEL. Shaywitz et al. [33] and Goldenring et al. [56] found greater activity and decreased habituation in a rodent model following administration of mixtures at doses near the ADIs. These mixture doses are in the range of doses in human mixture studies. Doses used in the human mixture studies were designed to mimic actual exposures in children.

A broad range of potential mechanisms by which the synthetic food dyes may impact behavior in susceptible children have been proposed. Additional research is warranted including:

- Animal testing in immature animals that includes a within-subjects design and measures of neurobehavior more similar to those in the human studies.
- Studies of the toxicokinetics of food dyes in humans and animals using modern techniques and including exposures during different life stages.
- Mechanistic studies and studies of underlying genetic susceptibility.
- Additional adequately powered clinical trials in children of the FD&C batch-certified synthetic food dyes with a cross-over, placebo-controlled, double blinded design utilizing validated outcome measures, inclusion of behavioral assessments by parents, and objective tests of attention and other behavioral measures by trained psychometricians. Such studies should attempt to evaluate whether the response differs by age, gender, ethnicity, race, or socioeconomic status through a design that evaluates dosing on a mg/kg/day basis.
- Studies that evaluate the potential long-term impacts of repeated exposures to food dyes in children.

Such research would provide additional data to inform appropriate acceptable daily intakes that explicitly protect children from neurobehavioral effects. In the short-term, the neurobehavioral effects of synthetic food dyes in children should be acknowledged and steps taken to reduce exposure to these dyes in potentially susceptible children.

## Supplementary Information

**Additional file 1: Table A.1.** Search Strategy. This table illustrates the literature search strategy. **Table A.2**. Clinical trials of synthetic food dyes and neurobehavioral outcomes in children: study details. This table provides study details for the 25 challenge studies in children reviewed by OEHHA. **Table A.3**. Clinical trials of synthetic food dyes and neurobehavioral outcomes: coding. This table provides the variables and coding used in the study quality analysis. **Table A.4**. Coding dictionary. This table defines the variables and numerical codes used in the study quality evaluation. **Table A.5**. Individual dyes. Developmental and adolescent/adult studies. This table provides study details of the animal toxicology studies of individual dyes reviewed by OEHHA. **Table A.6**. Dye mixtures. Developmental and adolescent/adult studies. This table provides study details of the animal toxicology studies of dye mixtures reviewed by OEHHA

## Data Availability

As this is a review, data sharing is not applicable to this article as no datasets were generated during the current study. Details of the studies we reviewed are contained in the supplementary tables. The study quality review and coding are available in the supplementary files. Exposure estimates were based on the National Health and Nutrition Examination Survey conducted in 2015 and 2016: CDC. 2017. NHANES 2015–2016 Demographics Data. Available: https://wwwn.cdc.gov/nchs/nhanes/search/datapage.aspx?Component=Demographics& CycleBeginYear = 2015: CDC. 2018. NHANES Dietary Data. Available: https://wwwn.cdc.gov/nchs/nhanes/Search/DataPage.aspx?Component=Dietary. CDC. 2019. National Health and Nutrition Examination Survey. Available: https://www.cdc.gov/nchs/nhanes/index.htm.

## Abbreviations

ADHD: Attention deficit hyperactivity disorder
ADI: Acceptable daily intake
CRF: Corticotrophin releasing factor
DNT: Developmental neurotoxicology
FDA: US Food and Drug Administration
FDA ADI NOAEL: The NOAEL used by FDA to derive the current FDA ADI
FD&C: Food Drug and Cosmetic Act, referring to dyes that must be batch-certified per FDA regulations
FAO: Food and Agriculture Organization of the World Health Organization
JECFA: Joint FAO/WHO Expert Committee on Food Additives
LOAEL: lowest-observed-adverse-effect level in a study
Mg: Milligrams
Mg/kg/day: Mg of substance per kg body weight per day
MAO: Monoamine oxidase
NIH: National Institutes of Health
NTP: National Toxicology Program
NHANES: National Health and Nutrition Examination Survey
NOAEL: No-observed-adverse-effect level in a study
OEHHA: Office of Environmental Health Hazard Assessment, California Environmental Protection Agency
OHAT: Office of Health Assessment and Translation
PND: Postnatal day
RCT: Randomized clinical trial
RCDP: Clinical trials that are randomized cross-over design, double-blinded and placebo controlled
UK: United Kingdom
US FDA: United States Food and Drug Administration
