# Supplementary material for: Potential impacts of synthetic food dyes on activity and attention in children: a review of the human and animal evidence
Source: Environ Health. 2022 Apr 29;21:45. doi: 10.1186/s12940-022-00849-9 (PMC9052604; doi:10.1186/s12940-022-00849-9)
Supplement: Supplementary file 1 — Additional file 1: Table A.1. Search Strategy. This table illustrates the literature search strategy. Table A.2. Clinical trials of synthetic food dyes and neurobehavioral outcomes in children: study details. This table provides study details for the 25 challenge studies in children reviewed by OEHHA. Table A.3. Clinical trials of synthetic food dyes and neurobehavioral outcomes: coding. This table provides the variables and coding used in the study quality analysis. Table A.4. Coding dictionary. This table defines the variables and numerical codes used in the study quality evaluation. Table A.5. Individual dyes. Developmental and adolescent/adult studies. This table provides study details of the animal toxicology studies of individual dyes reviewed by OEHHA. Table A.6. Dye mixtures. Developmental and adolescent/adult studies. This table provides study details of the animal toxicology studies of dye mixtures reviewed by OEHHA [file 12940_2022_849_MOESM1_ESM.docx]

**Additional Material - Miller et al, submitted**

**Table A.1 Search Strategy**

The following illustrates the search strategy.

| **ID** | **Key Word** | **Terms** |
| --- | --- | --- |
| 1 | (food coloring agents[mh] OR food[mh] OR food[tiab] OR foods[tiab] OR foodstuff*[tiab] OR beverage*[tiab] OR pharmaceutical*[tiab] OR medication*[tiab] OR dietary exposure[mh]) | Diet, Food & Generic food color terms |
| 2 | (Erythrosine[tiab] OR “Erythrosin”[tiab] OR “2',4',5',7'-Tetraiodofluorescein”[tiab] OR “2,4,5,7-Tetraiodofluorescein disodium salt”[tiab] OR “F D and C 3”[tiab] OR “Red No. 3”[tiab] OR “FDC Red 3”[tiab] OR “FD&C Red 3”[tiab] OR “1427 Red”[tiab] OR “1671 Red”[tiab] OR “9-(o-Carboxyphenyl)-6-hydroxy-2,4,5,7-tetraiodo-3H-xanthene-3-one disodium salt monohydrate”[tiab] OR “Aizen Food Red 3”[tiab] OR “C.I. 45430”[tiab] OR “C.I. 773”[tiab] OR “Acid Red 51”[tiab] OR “Food Red 14”[tiab] OR “Cerven kysela 51”[tiab] OR “Cerven potravinarska 14”[tiab] OR “Cilefa Pink B”[tiab] OR “E 127”[tiab] OR E127[tiab] OR “Food Color Red 3”[tiab] OR “Food Dye Red 3”[tiab] OR “Food Red 14”[tiab] OR “Food Red 3”[tiab] OR “Hexacert Red No. 3”[tiab] OR “LB-Rot 1”[tiab] OR “New Pink Bluish Geigy”[tiab] OR “Schultz No. 887”[tiab] OR “Tetraiodofluorescein sodium salt”[tiab] OR “Usacert Red No. 3”[tiab] OR 16423-68-0[rn]) | Erythrosine terms |
| 3 | (Tartrazine[mh] OR tartrazine[tiab] OR "yellow no 5"[tiab] OR "yellow 5"[tiab] OR 12225-21-7[rn] OR 1934-21-0[rn] OR e102[tiab] OR "e-102"[tiab]) | Tartrazine terms |
| 4 | ("sunset yellow" OR "ci 15-985"[tiab] OR "yellow no 6"[tiab] "yellow 6"[tiab] OR gelborange[tiab] OR "yellow 3"[tiab] OR "l-orange 2"[tiab] OR "orange no 2"[tiab] OR "e 110"[tiab] OR e110[tiab] OR 2783-94-0[rn] OR 1325-37-7[rn] OR 220-491-7[rn] OR 215-393-6[rn]) | Sunset Yellow terms |
| 5 | (Allura Red AC Dye [Supplementary Concept] OR "ci 16035"[tiab] OR "red 40"[tiab] OR "red no 40"[tiab] OR "r-40"[tiab] OR "curry red"[tiab] OR "food red 17"[tiab] OR "fancy red"[tiab] OR e129[tiab] OR "e-129"[tiab] OR "ccris 3493"[tiab] OR "hsdb 7260"[tiab] OR 25956-17-6[rn]) | Allura Red terms |
| 6 | (citrus red No. 2 [Supplementary Concept] OR "citrus red 2"[tiab] OR "solvent red no 80"[tiab] OR "solvent red 80"[tiab] OR "C.I. 12156"[tiab] OR "CI 12156"[tiab] OR E121[tiab] OR "e-121"[tiab] OR 6358-53-8[rn] OR 228-778-9[rn]) | Citrus Red terms |
| 7 | (Fast Green FCF [Supplementary Concept] OR "fast green"[tiab] OR "food green 3"[tiab] OR "food green no 3"[tiab] OR "solid green fcf"[tiab] OR "fd & c green no 3"[tiab] OR "fd & c green 3"[tiab] OR "FD and C green no 3"[tiab] OR "FD and C green c"[tiab] OR "ci 42053"[tiab] OR "c.i. 42053"[tiab] OR E143[tiab] OR "e-143"[tiab] OR 2353-45-9[rn] OR 219-091-5[rn]) | Fast Green terms |
| 8 | (indigo carmine[mh]OR "indigo carmine"[tiab] OR "D and C blue no 6"[tiab] OR "D and C blue 6"[tiab] OR "FD and C blue no 2"[tiab] OR "FD and C blue 2"[tiab] OR "FD & C blue no 2"[tiab] OR "FD & C blue 2"[tiab] OR "indigo blue"[tiab] OR "indigo disulfonate"[tiab] OR indigotin[tiab] OR indigotindisufonate[tiab] OR "acid blue 74"[tiab] OR indigocarmin*[tiab] OR "food blue no 2"[tiab] OR "food blue 2"[tiab] OR "amacid brilliant blue"[tiab] OR "food blue 1"[tiab] OR "food blue no 1"[tiab] OR "natural blue 2"[tiab] OR "natural blue o 2"[tiab] OR "grape blue a"[tiab] OR "airedale blue IN"[tiab] OR "acid blue w"[tiab] OR "cilefa blue r"[tiab] OR "intense blue"[tiab] OR "edicol supra blue x"[tiab] OR e132[tiab] OR e-132[tiab] OR 860-22-0[rn] OR 212-728-8[rn]) | Indigo Carmine terms |
| 9 | (brilliant blue [Supplementary Concept] OR "brilliant blue fcf"[tiab] OR "acid blue 9"[tiab] OR "acid blue no 9"[tiab] OR "blue 4"[tiab] OR "blue no 4"[tiab] OR "blue 1"[tiab] OR "blue no 1"[tiab] OR "c.i. 42090"[tiab] OR "ci 42090"[tiab] OR "caries check blue"[tiab] OR "d and c blue no 4"[tiab] OR "d and c blue 4"[tiab] OR "dc blue no 4"[tiab] OR "dc blue 4"[tiab] OR erioglaucine[tiab] OR "FD and C blue no 1"[tiab] OR "FD and C blue 1"[tiab] OR "FD & C blue no 1"[tiab] OR "FD & C blue 1"[tiab] OR e133[tiab] OR "e-133"[tiab] OR 3844-45-9[rn] OR 2650-18-2[rn]) | Brilliant Blue FCF terms |
| 10 | ("orange b"[tiab] OR "c.i. acid orange 137"[tiab] OR "ci acid orange 137"[tiab] OR 53060-70-1[rn]) | Orange B terms |
| 11 | ("sulfanilic acid"[tiab] OR sulfanilic acids[mh] OR benzidines[mh] OR benzidine[tiab] OR aminopyrazalone[tiab] OR 1-amino-2-naphthol-6-sulfonic acid [Supplementary Concept] OR "5-sulfoanthranilic acid"[tiab] OR "p-acetamidobenzene-sulfonic acid"[tiab] OR "1-amino-2-naphthyl sulfate"[tiab] OR "Cresidine-4-sulfonic acid"[tiab] OR "Naphthionic acid"[Supplementary Concept] | Metabolite terms |
| 12 | (#2 OR #3 OR #4 OR #5 OR #6 OR #7 OR #8 OR #9 OR #10 OR #11) | Combine Food Color Terms |
| 13 | #1 AND #12 | Colors + Food Terms |
| 14 | (erythrosine[ti] OR tartrazine[ti] OR sunset yellow[ti] OR “allura red”[ti] OR "citrus red no 2"[ti] OR “fast green”[ti] OR “indigo carmine”[ti] OR "brilliant blue fcf"[ti] OR "orange b"[ti] OR "acid orange 137"[ti]) | Food colors - title only |
| 15 | (neurobehav*[tiab] OR neurodevel*[tiab] OR neurocognit*[tiab] OR neurotoxic*[tiab] OR neurolog*[tiab] OR neurobiochemi*[tiab] OR neuropsych*[tiab] OR nerve[tiab] OR nervous[tiab] OR neural[tiab] OR brain[tiab] OR forebrain[tiab] OR midbrain[tiab] OR hindbrain[tiab] OR hippocampus[tiab] OR "prefrontal cortex"[tiab] OR “frontal cortex”[tiab] OR “frontal lobe”[tiab] OR “parietal lobe”[tiab] OR “temporal lobe”[tiab] OR “occipital lobe”[tiab] OR cerebellum[tiab] OR cogniti*[tiab] OR behavior*[tiab] OR memory[tiab] OR motor*[tiab] OR attention[tiab] OR adhd[tiab] OR hyperactiv*[tiab] OR activity[tiab] OR inattenti*[tiab] OR neurodevelopmental disorders[mh] OR hyperkine*[tiab] OR hyperkinesis[mh] OR nervous system diseases[mh] OR nervous system/drug effects[mh] OR "behavior and behavior mechanisms"[mh] OR mental disorders[mh] OR autism spectrum disorder[mh] OR autis*[tiab] OR 'conduct disorder'[tiab] OR substance-related disorders[mh] OR substance abuse*[tiab] OR drug abuse*[tiab] OR alcohol abuse*[tiab] OR alcoholi*[tiab] OR intoleran*[tiab] OR aggressi*[tiab] OR violen*[tiab]) | Neuro Outcome terms |
| 16 | (risk[mh] OR risk assessment[mh] OR risk[tiab] OR risks[tiab] OR expos*[tiab] OR intake[tiab] OR consumption[tiab] OR consumed[tiab] OR consumes[tiab] OR ingest*[tiab] OR dose[tiab] OR doses[tiab] OR maternal exposure[mh] OR paternal exposure[mh] OR prenatal exposure delayed effects[mh] OR dietary exposure[mh] OR perinatal[tiab] OR in utero[tiab] OR pregnancy[tiab]) | Exposure terms |
| 17 | (pharmacology[mh] OR pharmacology[sh] OR mechanism*[tiab] OR pathway*[tiab] OR 'signal transduction'[mh] OR signal*[tiab] OR epigenomics[mh] OR epigenesis, genetic[mh] OR epigenetic*[tiab] OR immunosupressive agents[mh] OR immun*[tiab] OR immunotoxins[mh] OR reactive oxygen species[mh] OR pharmacokinetics[mh] OR pharmacokinetic*[tiab] OR toxicokinetic*[tiab] OR oxidative stress[mh] OR inflammation[mh] OR immune evasion[mh] OR apoptosis[mh] OR apoptosis[tiab] OR 'programmed cell death'[tiab] OR 'cell proliferation[mh] OR 'receptors, cytoplasmic and nuclear'[mh] OR toxicity[mh] or 'receptor mediated'[tiab]) | Mechanism terms |
| 18 | #14 AND (#15 OR #16 OR #17) | Color in Title + Outcomes |
| 19 | #13 OR #18 | Final set |

##### Table A.2 Clinical trials of synthetic food dyes and neurologic outcomes in children: study details

| **Study**  **Location**  **Demographics** | **Recruitment and design** | **Exposure** | **Exposure method** | **Outcome** | **Outcome method** | **Main results** | **Other results** | **Other aspects of causal inference** | **Notes** |
| --- | --- | --- | --- | --- | --- | --- | --- | --- | --- |
| Adams, 1981  Location: US  Hyperactive: yes  Responders: yes  Ages: 4-11  N: 18 | Cohort: announcement in Feingold Association newsletter  Selection: NA  Recruitment: unclear  Participation: unclear  Cross-over: yes  Randomized: yes  Blinded: double  Placebo: yes  Adequate placebo: NA | Yellow No. 5, red No. 3, red No. 40 and yellow No. 7  Daily dose: 26.3 mg | On elimination diet: likely  Regimen: given in cupcake or lemonade 3-4 hours before outcome assessment  Placebo: Feingold cupcake and lemonade  Vehicle: cupcake and lemonade  Washout: elimination diet, period unclear (given at “second appointment”)  Infractions: monitored | Activity levels, fine and gross motor skills, auditory and visual memory, parent ratings (not described) | Method: Auditory memory (McCarthy Scales), visual memory (Illinois Test of Psycholinguistic Ability), receptive language (Peabody Picture Vocabulary Test), parental observations  Validated: unclear  Timing: 3-4 hours after the snack | **Elimination diet:** NA  **Challenge:**  Parent: no “significant differences”  Teacher: NA  Other: no “significant differences”  Nine of the 14 outcomes variables showed a tendency towards increased symptoms for the active challenge but effect sizes were described as “slight” (p=0.40). Actual results not provided | Timing: NA  Age effect: not seen  Order effect: not seen  Individual results: not given  Replication: not done | Magnitude (>20%): no  Statistical significance: no  Dose-response: NA  Subgroup only: no  Funder: no information  Reported conflicts: no information  Full results: actual test results not provided | - All children had Conners scores ≥15 prior to starting Feingold diet |
| Bateman et al., 2004  Location: UK  Hyperactive: mixed  Responders: no  Ages: 3  N: 277 | Cohort: all 2,878 children on the Isle of Wight  Selection: all  Recruitment: unclear  Participation: 70%  Cross-over: yes  Randomized: yes  Blinded: double  Placebo: yes  Adequate placebo: drinks could not be differentiated in blind testing (no actual data given) | Elimination diet  Sunset yellow, tartrazine, carmoisine, ponceau 4R (5 mg each), and 45 mg sodium benzoate  Daily dose: 20 mg | On elimination diet: yes  Regimen: four week study: weeks two and four received daily placebo or food dye in fruit drink, washout periods in between  Placebo: juice without the active challenge agents  Vehicle: juice  Washout: elimination diet, one week between active challenge and placebo  Infractions: 66% had at least 1 mistake, only 8% had ≥6 mistakes, 81% of children drank all the challenge or placebo drinks | Hyperactivity; others | Method: weekly observation of free play, bear and dragon task, hiding stickers task, draw a line slowly and walk a line slowly – validated per authors; daily Weiss-Werry-Peters items (parents)  Validated: yes  Timing: weekly clinic visits with research psychologist and daily parent ratings | **Elimination diet:**  Parent: reduction in hyperactivity scores (p<0.001). Effect size appears >10% based on their Figure 3  Teacher: NA  Other: no effect  **Challenge:**  Parent: increased hyperactivity (p<0.02)  Teacher: NA  Other: no effect  No interaction by prior hyperactivity or atopy | Timing: NA  Age effect: NA  Order effect: not seen  Individual results: not given  Replication: not done | Magnitude (>20%): yes  Statistical significance: yes  Dose-response: NA  Subgroup only: no  Funder: UK Food Standards Agency and the South West Regional Research and Development Directorate. Smith Kline Beecham contributed to the challenge materials  Reported conflicts: no information  Full results: yes | - Atopy based on skin prick testing - Initial hyperactivity based on EAS activity scale and Weiss-Werry-Peters Activity Scale - No interaction with atopy or prior hyperactivity - Standardized effect size: 0.39 |
| Conners et al., 1976  Location: US  Hyperactive: yes  Responders: no  Ages: 6-12  N: 15  Elimination diet study | Cohort: unclear  Selection: unclear  Recruitment: unclear  Participation: 15/37 = 40.5%  Cross-over: yes  Randomized: yes  Blinded: unclear  Placebo: yes  Adequate placebo: NA | Feingold diet  Daily dose: NA | On elimination diet: NA  Regimen: following a 2 week baseline period, participants given Feingold diet and control diet for two weeks in random order  Placebo: control diet (described in the articles Appendix)  Vehicle: NA  Washout: none, no time between Feingold and control diets  Infractions: infractions per week were 1.5 for the control diet and 1.33 for the elimination diet | Hyperkinesis | Method: Conners rating scales, hyperkinesis index score ≥15 by parents and teachers – frequency not clear; global assessment score by the researchers based on parent/teacher ratings and parent interview at end of each 2 weeks  Validated: yes  Timing: weekly | **Elimination diet:**  Parent: reductions of about 15% in hyperkinesis scores with elimination vs. control diet but not statistically significant  Teacher: similar reductions but statistically significant (p<0.005)  Other: greater improvement on the global score on the Feingold than the control diet (p=0.01, one tailed)  **Challenge:** NA | Timing: NA  Age effect: NA  Order effect: yes, changes much greater with control diet first  Individual results: not given  Replication: not done | Magnitude (>20%): borderline  Statistical significance: yes  Dose-response: NA  Subgroup only: no  Funder: no information  Reported conflicts: no information  Full results: yes | - Experimental diet involved removal of natural salicylates, synthetic colors and flavors, control diet did not. Eliminated foods and the control diet described in the articles appendix |
| Conners et al., 1980  Location: US  Hyperactive: yes  Responders: yes  Ages: 5-10  N: 9 | Cohort: responders to elimination diet and blind challenges in previous trials  Selection: unclear  Recruitment: unclear  Participation: unclear  Cross-over: yes  Randomized: yes  Blinded: double  Placebo: yes  Adequate placebo: NA | Multiple Synthetic colors  Daily dose: 15 mg | On elimination diet: yes  Regimen: two sessions at 1-2 week intervals, two chocolate cookies given at the beginning of each session  Placebo: cookie without the active challenge  Vehicle: chocolate cookie  Washout: on elimination diet for 1-2 weeks between challenge and placebo  Infractions: monitored intakes | Activity levels, behavior ratings, attention and learning | Method: actometer, chair motion detector, study specific behavioral ratings, attention and learning task designed by Swanson and Kinbourne  Validated: unclear  Timing: observations at baseline and 45, 90, 135, and 180 minutes after dosing | **Elimination diet:** NA  **Challenge:**  Parent: NA  Teacher: NA  Other: activity testes and observer ratings don’t seem to differ between active challenge and placebo (shown in figure form). Learning errors appear worse with active challenge but not consistent across the two sessions | Timing: NA  Age effect: NA  Order effect: not seen  Individual results: not given  Replication: not done | Magnitude (>20%): no  Statistical significance: no  Dose-response: NA  Subgroup only: no  Funder: NIH  Reported conflicts: no information  Full results: yes | - Possible practice effect may have masked some findings - synthetic colors not described in detail |
| David et al., 1987  Location: UK  Hyperactive: no  Responders: yes  Ages: 1-12  N: 24 | Cohort: referred to allergy clinic, previous adverse behavioral reaction to food additives  Selection: all children  Recruitment: 24/30 = 80.0%  Participation: 100%  Cross-over: yes  Randomized: no  Blinded: double (see notes)  Placebo: yes  Adequate placebo: NA | Tartrazine  Daily dose: 50 and 250 mg | On elimination diet: yes  Regimen: given orange juice or Ribena throughout. Single dose of 50 mg, followed at least 2 hours later by a single dose of 250 mg. Both in either orange juice or Ribena (which contains sodium benzoate). Benzoic acid challenge given after tartrazine challenge on a separate day  Placebo: Orange juice or Ribena  Vehicle: Orange juice or Ribena  Washout: all subjects were on elimination diets at the time of the study, it appears the time before the study was the comparison period  Infractions: monitored | Any behavioral change following dye administration | Method: observation by parent and nursing staff for “any change in the child’s behavior” for an unclear period  Validated: no  Timing: unclear | **Elimination diet:** NA  **Challenge:**  Parent: No behavioral change in any child for placebo or active. No change upon return to “normal diet”  Teacher: NA  Other: Same as parent | Timing: NA  Age effect: NA  Order effect: NA  Individual results: yes, but no effects seen  Replication: not done | Magnitude (>20%): no  Statistical significance: no  Dose-response: not seen  Subgroup only: no  Funder: no information  Reported conflicts: no information  Full results: yes | - 19 boys and 5 girls - Six children had attention deficit disorder - Challenges were performed while participants were in the hospital: 12 inpatients and 12 outpatients - Benzoic acid also tested - Tartrazine challenge done first, benzoic acid challenge done a few days later. - Parents or observers did not know whether the child was receiving tartrazine or benzoic acid |
| Goyette et al., 1978  Location: US  Hyperactive: yes  Responders: yes  Ages: 4-12  N: 16 | Cohort: unclear  Selection: unclear  Recruitment: unclear  Participation: 16/27 = 59%  Cross-over: yes  Randomized: unclear  Blinded: double  Placebo: yes  Adequate placebo: NA | Elimination diet  “all synthetic colors currently approved by the FDA”  Daily dose: 26 mg (see notes) | On elimination diet: yes  Regimen: two challenge or placebo items per day in 2 week alternating sequences over 8 weeks  Placebo: cookie without the active challenge ingredient  Vehicle: chocolate cookie  Washout: none  Infractions: NA | Hyperkinesis; visual motor tracking | Method: Conners Parent/Teacher Hyperkinesis Index; Zero Input Tracking Analyzer and Auxillary Distraction Task (ZITA/ADT)  Validated: yes  Timing: 3 times per week (Conners); 1-2 hours after ingestion (ZITA/ADT) | **Elimination diet:**  Parent: 57% reduction in behavioral problems (no p-value)  Teacher: 34% reduction in behavioral problems (no p-value)  Other: no results given for ZITA/ADT  **Challenge:**  Parent: Initially, no effects. Second study (N=13) with parent rating 1-3 hours after challenge showed challenge effect (p<0.025) (standardized effect size = 0.38)  Teacher: no effects  Other: performance deficits on ZITA/ADT but not statistically significant and effect size not given | Timing: effects seen within one hour of challenge but not 2-3 hours after  Age effect: greater response in younger children  Order effect: not seen  Individual results: 3 children with large challenge effect on attention tests  Replication: similar results when repeated in 3 responders | Magnitude (>20%): yes  Statistical significance: yes  Dose-response: NA  Subgroup only: no  Funder: no information  Reported conflicts: no information  Full results: no, some effect sizes not given | - Information on dose, participation, and some effect sizes given in C.K. Conners 1980, Food Additives and Hyperactive Children, Prenum Press, New York, pages 41-68 |
| Harley et al., 1978a  Location: US  Hyperactive: mixed  Responders: no  Ages: 3-13  N: 80 (see notes)  Elimination diet study | Cohort: referred to researchers hospital for hyperactivity  Selection: unclear  Recruitment: unclear  Participation: unclear  Cross-over: yes  Randomized: yes  Blinded: double  Placebo: yes  Adequate placebo: parents were not able to identify diet cross-overs | Feingold diet vs. control  Daily dose: NA | On elimination diet: NA  Regimen: each diet used for 3-4 weeks  Placebo: control diet, not well described  Vehicle: NA  Washout: unclear  Infractions: 0.65-1.33 deviations per week per teachers and parent reports | Hyperactivity | Method: neuropsychological testing and laboratory and classroom observations; parent and teachers Conners P-TQ  Validated: yes  Timing: neuropsychological testing and laboratory observations at baseline and conclusion of each 3-4 week diet period; classroom observations 3 times per week; parent and teachers Conners scores weekly | **Elimination diet:**  *In older children (ages 6-13 years)*  Parent: 13 of 36 (36%) rated as improved on elimination diet, 6 worsened (17%), 17 no change (p<0.05)  Teacher: 6 of 36 improved, 10 worsened, and 20 unchanged (p >0.05)  Other: no effect of diet on not attending to task, restless motor activity, locomotor activity, or classroom disruption; elimination diet better for one neuropsychological test but worse for several others  *In preschool children:*  Parent: all 10 improved on the elimination diet  Teacher: NA  Other: no diet effect seen but few details given  **Challenge:** NA | Timing: NA  Age effect: stronger effects appear to be seen in younger subjects (3-6 years)  Order effect: greater effects in those receiving control diet first in older boys, no order effect in preschool boys  Individual results: yes (see results)  Replication: not done | Magnitude (>20%): yes  Statistical significance: yes  Dose-response: NA  Subgroup only: no  Funder: no information  Reported conflicts: no information  Full results: no, limited results given for preschoolers | - All boys - Included 36 older hyperactive boys (ages 6-13 years) and 34 matched controls, and 10 hyperactive preschool boys (ages 3-6 years) - Medications for hyperactivity were terminated - Feingold diet: foods with added salicylates, synthetic food dyes, and synthetic flavors were eliminated. Control diet not well described - Classroom observations included controls without hyperactivity matched on classroom, age, grade, teachers judgement of academic ability |
| Harley et al., 1978b  Location: US  Hyperactive: mixed  Responders: yes  Ages: 3-12  N: 18 | Cohort: previous responders and matched controls  Selection: unclear  Recruitment: unclear  Participation: unclear  Cross-over: yes  Randomized: unclear  Blinded: double  Placebo: yes  Adequate placebo: none of the parents or children identified placebo vs. challenge | 27 mg of food colors per item, 2 items per day  Daily dose: 54 mg | On elimination diet: yes  Regimen: two periods of placebo or challenge materials for 2-3 weeks each  Placebo: vehicle without the food dyes  Vehicle: cookies or candy bars  Washout: none  Infractions: limited data, maximum number was 6 over 11 weeks in one subject | Hyperactivity; deviant behavior, gross motor activity, non-work, disturbing behavior, isolation, on and off task activity (attention) | Method: Conners 10-item P-TQ to parents and teachers two times per week for 13 weeks; classroom observation by trained observers using the Werry and Quay method two times per week for 13 weeks; neuropsychological exams at baseline and end of each diet period; for classroom observations  Validated: yes  Timing: two times per week | **Elimination diet:** NA  **Challenge:**  Parent: no group effect  Teacher: no group effect  Other: no group effect on classroom behavior or neuropsychological testing | Timing: NA  Age effect: NA  Order effect: a challenge effect seems to be seen when placebo given first (their Figure 1)  Individual results: yes, one subject seemed to show a challenge effect on parent rating and classroom observation  Replication: not done | Magnitude (>20%): unclear  Statistical significance: no  Dose-response: NA  Subgroup only: no  Funder: University of Wisconsin Food Research Institute, Nutrition Foundation  Reported conflicts: no information  Full results: yes | - Controls matched to responders on sex, grade, and academic ability - No child was on medications - Challenge materials not fully described |
| Levy and Hobbes, 1978  Location: Australia  Hyperactive: yes  Responders: yes  Ages: mean age 5 years and 2 months (range not given)  N: 8 | Cohort: unclear  Selection: unclear  Recruitment: unclear  Participation: 7/8 = 87%  Cross-over: yes  Randomized: yes  Blinded: unclear  Placebo: yes  Adequate placebo: mothers could not differentiate challenge from placebo | Tartrazine  Daily dose: 4 mg | On elimination diet: likely  Regimen: attempted to replicate procedures in Goyette et al. (unpublished) but few details provided. It appears that 4 challenge or placebo cookies were given each day for 14 days each  Placebo: cookie without extra tartrazine  Vehicle: cookie  Washout: unclear  Infractions: NA | Hyperactivity | Method: Conners scale, parent  Validated: yes  Timing: unclear | **Elimination diet:** NA  **Challenge:**  Parent: 2.6 points higher (13%) during the challenge but result not statistically significant  Teacher: NA  Other: NA | Timing: NA  Age effect: NA  Order effect: NA  Individual results: not given  Replication: not done | Magnitude (>20%): no  Statistical significance: no  Dose-response: NA  Subgroup only: no  Funder: no information  Reported conflicts: no information  Full results: no, limited information on the outcome metrics | - 7 boys and 1 girl |
| Levy et al., 1978  Location: Australia  Hyperactive: yes  Responders: no  Ages: 4-8  N: 22 | Cohort: referred for hyperactivity or over-activity, distractibility, and impulsive and aggressive behavior  Selection: unclear  Recruitment: unclear  Participation: unclear  Cross-over: yes  Randomized: unclear  Blinded: mixed (see notes)  Placebo: yes  Adequate placebo: challenge and placebo biscuits were not identical in appearance | Feingold diet  Tartrazine  Daily dose: 5 mg | On elimination diet: yes  Regimen: given daily challenge or placebo for 2 weeks each  Placebo: biscuit without tartrazine  Vehicle: biscuits  Washout: none  Infractions: average of 1-2 per child during challenge-placebo period | Hyperactivity, attention, IQ, and multiple others | Methods: Conners P-TQ (hyperactivity) by mother, teacher, psychologist; Spraque Ballistographic Chair (motility); Continuous Performance Test (attention); Draw a line slowly test (impulsivity); Jean Ayres tests (perceptual motor functioning); Illinois Test of Pscycho-Linguistic Ability (memory); Wechsler (IQ) performed at beginning of trial and approximately 2 week intervals  Validated: yes  Timing: Conners done at baseline and after 4 weeks of placebo plus challenge, some other tests at baseline and after 4 week washout period. Other intervals not well described | **Elimination diet:**  Parent: improved scores (p<0.005), actual scores not given  Teacher: no effect  Other: no effect  **Challenge:**  Parent: no effect overall; a challenge effect was seen (p<0.025) in the 13 children meeting criteria of Goyette (see notes)  Teacher: no effect  Other: no effect for other tests or clinicians scores  Similar results when analyses confined to the 16 children with the highest hyperactivity scores except positive result on Mazes subtest of the WISC (p<0.025) (actual results not given) | Timing: NA  Age effect: NA  Order effect: NA  Individual results: not given  Replication: not done | Magnitude (>20%): unclear  Statistical significance: yes  Dose-response: NA  Subgroup only: yes, those meeting Goyette criteria  Funder: National Health and Medical Research Council  Reported conflicts: no information  Full results: no, mostly just p-values given | - 19 boys and 3 girls - Blinding: it appears the initial elimination diet was not blind but the challenge vs. placebo may have been double blinded but this is unclear - Goyette criteria: <8 years old, ≥10 on Conners scale, ≥12% reduction in mothers rating after elimination diet for 1 month |
| Lok et al., 2013  Location: Hong Kong  Hyperactive: no  Responders: no  Ages: 8-9  N: 130 | Cohort: selected schools in Hong Kong  Selection: all  Recruitment: 3.3%  Participation: 130/175 = 74.3%  Cross-over: yes  Randomized: yes  Blinded: double  Placebo: yes  Adequate placebo: NA | Sunset yellow, carmoisine, tartrazine, and Ponceau 4R  Daily dose: 62.4 mg | On elimination diet: yes  Regimen: elimination diet for 6 weeks then 1 week each of synthetic food coloring, sodium benzoate, or placebo in random order with one week washout period between  Placebo: lactose  Vehicle: capsule  Washout: one week on elimination diet  Infractions: 80% consumed ≥85% of the capsules, 86.2% had no reported dietary mistakes | ADHD symptoms and behavior | Methods: Strengths and Weaknesses of ADHD Symptoms and Normal Behaviors (SWAN) rating scale (parents and teachers); Child Behavior Checklist (CBCL) (teachers only)  Validated: yes  Timing: weekly SWAN; CBCL-unclear | **Elimination diet:** NA  **Challenge:**  Parent: no effect  Teacher: no effect  Other: NA  Similar results in those who consumed ≥85% of the capsules  No effect with sodium benzoate | Timing: NA  Age effect: NA  Order effect: NA  Individual results: not given  Replication: not done | Magnitude (>20%): no  Statistical significance: no  Dose-response: NA  Subgroup only: no  Funder: Centre for Nutritional Studies, The Chinese University of Hong Kong  Reported conflicts: none declared  Full results: yes | - 70 boys and 60 girls - Children with ADHD excluded - Effect sizes: for children taking 85% or more of the challenge capsules, the effect sizes were .07 for CBCL score and .01 for SWAN - Benzoic acid also tested - Further details on doses of each dye provided |
| Mattes and Gittleman, 1981  Location: US  Hyperactive: mixed  Responders: yes  Ages: 4-13  N: 11 | Cohort: recruited from local chapters of the Feingold Association  Selection: unclear  Recruitment: unclear  Participation: 11/13 = 85%  Cross-over: yes  Randomized: yes  Blinded: double  Placebo: yes  Adequate placebo: the parents of the six children who showed a difference between active ingredient and placebo could not consistently guess the correct cookie type | US FDA approved synthetic food colorings in proportions thought to reflect normal patterns of consumption  Daily dose: 13-78 mg | On elimination diet: yes  Regimen: placebo or active challenge for one week each with one week washout in between. Started with 1 cookie per day on day one (13 mg) and increased one cookie each day  Placebo: cookies without synthetic dyes  Vehicle: cookies  Washout: one week  Infractions: 3 failed to eat maximum of 6 cookies (see results) | Hyperactivity, multiple others | Methods: Conners Ratings Scales (parents and teachers); study specific hyperactivity scale; psychiatric evaluation; Childrens Diagnostic Scale; distractibility test; psychologist rating of child’s behavior once or twice weekly  Validated: yes  Timing: all tests done at baseline and weekly; distractibility test given within 1.5 hours of ingestion; brief Conners test (teachers and parents) done day 3 and 5 of each period | **Elimination diet:** NA  **Challenge:**  Parent: no consistent effects  Teacher: no consistent effects  Other: no effects  Similar results in children getting full daily dose, in hyperactive, and in different age groups | Timing: NA  Age effect: none seen  Order effect: none seen  Individual results: yes, but results mixed and unclear  Replication: not done | Magnitude (>20%): no  Statistical significance: no  Dose-response: NA  Subgroup only: no  Funder: US Public Health Service  Reported conflicts: no information  Full results: yes | - Some children diagnosed as hyperactive (n=5) and some not - Exact ingredients in the active cookie not provided in the article - In a one week trial prior to challenges, 2 children who reacted adversely to placebo cookies were removed from the trial - Included only 1 child under 5 years old - Multiple doses given but dose-response not tested |
| Mattes and Gittleman-Klein, 1978  Location: US  Hyperactive: yes  Responders: yes  Ages: 10  N: 1 | Cohort: NA  Selection: NA  Recruitment: NA  Participation: NA  Cross-over: yes  Randomized: yes  Blinded: double  Placebo: yes  Adequate placebo: NA | All “commonly used synthetic food colorings”  Daily dose: 1/5^th^ the average US daily intake per cookie (unclear if for adults or children) | On elimination diet: yes  Regimen: Two trials. In the first, active challenge or placebo cookies given for 1 week each increasing from one to six cookies per day. In the second, 3 active or 3 placebo cookies per day on Wednesday and Thursday (same cookie each week) for 10 weeks  Placebo: cookie without synthetic dyes  Vehicle: cookie  Washout: 5 days on elimination diet  Infractions: NA | Hyperactivity | Methods: Conners questionnaire by parent, teacher, and child; mothers guess of cookie type based on child’s behavior  Validated: yes  Timing: likely weekly | **Elimination diet: NA**  **Challenge:**  Parent: all scores were low, mother guessed correct cookie type in 8 of 10 weeks (p=0.055), higher mean Conners score on active cookie (3.00 vs. 1.40) but not statistically significant. When data combined with those of a dose range finding study mother guessed correct cookie type in 9 of 11 trials (p=0.033)  Teacher: no effect  Child: no effect  Other: NA | Timing: NA  Age effect: NA  Order effect: NA  Individual results: yes (n=1)  Replication: dosing study followed by full trial | Magnitude (>20%): yes  Statistical significance: yes  Dose-response: NA  Subgroup only: no  Funder: no information  Reported conflicts: no information  Full results: yes | - A dose range finding study was also done in which the child was increased from 1 to six cookies per day. Stopped after third day due to restlessness and irritability. Parent ratings increased but no change in teachers scores - Actual doses where effects seen not given |
| McCann et al., 2007  Location: UK  Hyperactive: no  Responders: no  Ages: 3, 8-9  N: 153 (age 3) and 144 age (8-9) | Cohort: community based (playgroups, nurseries, and schools) throughout Southampton  Selection: unclear  Recruitment: about 17% (3 year olds) and 23% (8-9 year olds)  Participation: 90%  Cross-over: yes  Randomized: yes  Blinded: double  Placebo: yes  Adequate placebo: masked trial in 20 young adults showed drinks could not be differentiated | **Mix A:** 20 mg (3 year olds) and 25 mg (8-9 year olds) total of synthetic dyes including tartrazine  **Mix B:** 30 mg (3 year olds) and 64 mg (8-9 year olds) not including tartrazine  See notes  Daily dose: 20, 30, and 64 mg depending on mix and age | On elimination diet: yes  Regimen: six week trial with challenge or placebo on weeks 2, 4, and 6, and placebo on weeks 1, 3, and 5  Placebo: fruit juice without active challenge  Vehicle: fruit juice  Washout: none, it appears that weeks 1, 3, and 5 were included in the analyses but this is unclear (see notes)  Infractions: low rate of reported dietary infractions | Hyperactivity  Attention | Methods: overall standardized global hyperactivity aggregate (GHA) scores that combined weekly ADHD rating scale IV by teachers, weekly Weiss-Werry-Peters hyperactivity scale by parents, classroom observation by trained observers, and Conners continuous performance test II scores (only in 8-9 year olds)  Validated: yes  Timing: weekly | **Elimination diet:** NA  **Challenge:**  Parent: NA  Teacher: NA  Other: standardized differences in GHA scores in challenge vs. placebo  *Three year olds:*  Mix A: 0.20 (0.01 to 0.39)  Mix B: 0.17 (-0.03 to 0.36)  *8-9 year olds:*  Mix A: 0.08 (-0.02 to 0.17)  Mix B: 0.12 (0.03 to 0.22)  Somewhat higher effect sizes in high consumption groups and those with complete data  Evidence of moderation by histamine degradation gene polymorphisms HNMT T939C and HNMT Thr105Ile in 3 and 8/9-year-old children and by DAT1 polymorphism in 8/9-year-old children (Stevenson et al., 2010) | Timing: NA  Age effect: greater effect sizes in younger children (3 year olds)  Order effect: unclear  Individual results: greater variability in responses reported for Mix B in 3 year olds but few details given  Replication: not done | Magnitude (>20%): yes  Statistical significance: yes  Dose-response: NA  Subgroup only: no  Funder: UK Food Standards Agency  Reported conflicts: none declared  Full results: yes | - Similar numbers of boys and girls - Percentage receiving free lunches matched that of the city as a whole - Mix A: additives used in previous studies - Mix B: average daily food additives in UK children - Details on specific dyes used given in the article - Both mixes included sodium benzoate - Washout: no effect shown for the type of challenge (active or placebo) in the previous challenge period but results are “preliminary” and details not provided - Drop-outs unrelated to behavior problems - Effect size of 0.20 is about 10% of the behavioral score difference between children with and without ADHD - Little change in results with adjustment for week of study, baseline score, sex, pre-trial diet, maternal education, and social class |
| Pollock and Warner, 1990  Location: UK  Hyperactive: mixed  Responders: yes  Ages: 2-15  N: 19 | Cohort: pediatric allergy clinic and population survey of food additive intolerance  Selection: unclear  Recruitment: unclear  Participation: 19/39 = 49%  Cross-over: yes  Randomized: yes  Blinded: double  Placebo: yes  Adequate placebo: NA | Tartrazine 50 mg, sunset yellow 25 mg, carmoisine 25 mg, and amaranth 25 mg  Daily dose: 125 mg | On elimination diet: yes  Regimen: active capsule or placebo taken daily for 2-3 separate weeks during seven week trial  Placebo: lactose  Vehicle: capsules (opaque)  Washout: one week washout after each week of active challenge  Infractions: NA | Hyperactivity, allergic symptoms | Methods: Conners hyperactivity index, overall behavioral assessment (parents) and allergic symptoms collected from parents in daily questionnaires  Validated: yes  Timing: daily | **Elimination diet:** NA  **Challenge:**  Parent: higher Conners scores with active challenge vs. placebo (p<0.01). Group means not given but individual data shown in figure and table. Parents behavioral rating correct (worse with active challenge) 60/92 times (65%). No difference in allergic symptoms  Teacher: NA  Other: NA | Timing: similar effects seen day 1 vs. day 7  Age effect: NA  Order effect: not seen  Individual results: yes, at least some increase with active challenge seen in most children  Replication: not done | Magnitude (>20%): yes  Statistical significance: yes  Dose-response: NA  Subgroup only: no  Funder: Ministry of Agriculture, Fisheries, and Food  Reported conflicts: no information  Full results: yes | - One child diagnosed as being “hyperkinetic”, two children with Conners scores ≥15 - Four children were withdrawn after parents noted unacceptable behavioral changes early in the trial. Two of these children were taking active capsules and the other two were taking placebo |
| Rapp, 1978  Location: US  Hyperactive: yes  Responders: no  Ages: 5-16  N: 24 | Cohort: hyperactive and referred by physicians, psychologists, and members of the Association for Children with Learning Disabilities  Selection: unclear  Recruitment: unclear  Participation: unclear  Cross-over: yes  Randomized: yes  Blinded: yes  Placebo: yes  Adequate placebo: NA | Red, yellow, green, and blue McCormick’s food coloring  Daily dose: “three drops” or 0.1 ml total | On elimination diet: no  Regimen: dyes or control (grape juice) given once sublingually, each on the same day  Placebo: similarly colored grape juice  Vehicle: none  Washout: none  Infractions: monitored | Hyperactivity, other | Methods: direct observations of activity by trained “housewife” and study nurse; standard one-minute WISC Coding test and three-minute Ayres Southern California Motor Accuracy test, about 10-15 minutes before and after each challenge  Validated: unclear  Timing: within 10-15 minutes of dosing | **Elimination diet:** NA  **Challenge:**  Parent: NA  Teacher: NA  Other: moderate or marked increase in activity was observed in 9 of 24 patients (37%), a slight increase in five of 24 (21%), and no change in 10 of 24 (42%). The grape juice control caused a marked change in one child (4%), a slight change in two, and no change in 20 of 24 children. No effects on WISC and Ayres tests | Timing: NA  Age effect: NA  Order effect: NA  Individual results: yes, see results  Replication: mentioned but no results given | Magnitude (>20%): yes  Statistical significance: likely  Dose-response: NA  Subgroup only: no  Funder: American Academy of Pediatrics Memorial and Endowment Fund for Children.  Reported conflicts: no information  Full results: no, actual scores and test details not given | - 6 girls and 18 boys - 15 currently on medications, 8 discontinued them during the study - 14 of 19 had a family history of allergies - Elimination diet also tested but involved milk, wheat, eggs, cocoa, corn, sugar in addition to food colorings (results not reported here) |
| Rose, 1978  Location: US  Hyperactive: yes  Responders: yes  Ages: 8  N: 2 | Cohort: children with hyperactivity, not on medications, and using the KP diet for at least 4 months, “a search of the community”  Selection: unclear  Recruitment: unclear  Participation: unclear  Cross-over: yes  Randomized: no  Blinded: double  Placebo: yes  Adequate placebo: cookies with food color could not be identified vs. those without in a separate study | Tartrazine  Daily dose: 1.2 mg (0.05 mg/kg) | On elimination diet: yes  Regimen: one cookie was given each day for what appears to be 30 days, with tartrazine in the cookie on two of the days, placebo on two of the days, and elimination diet otherwise  Placebo: cookie with no dyes or natural salicylates  Vehicle: cookie  Washout: unclear, it appears that placebo may have followed the active challenge without a washout in some instances (see their Figures 1 and 2)  Infractions: 1-2 dietary infractions per subject | Hyperactivity, other | Methods: daily parent logs of “significant” changes in behavior and daily 30 minute observations by graduate students for the following variables: on task, out-of-seat, and physical aggression  Validated: observer reliability vs. standard data ranged from 82.4 to 100%  Timing: daily | **Elimination diet:** NA  **Challenge:**  Parent: “correlated perfectly” with observer data, but actual results not given  Teacher: NA  Other: observer data given in figure form seem consistent with a challenge effect. Statistically significant changes seen in out-of-seat and percent time on task for both subjects (all p-values <0.01). Effect sizes appear >20% based on figures. No association with aggressive behavior but rates were very low | Timing: NA  Age effect: NA  Order effect: NA  Individual results: yes, see results  Replication: not done | Magnitude (>20%): yes  Statistical significance: yes  Dose-response: NA  Subgroup only: no  Funder: no information  Reported conflicts: no information  Full results: no, actual results from the parents logs not given | - Both girls |
| Rowe and Rowe, 1994  Location: Australia  Hyperactive: mixed  Responders: mixed  Ages: 2-14  N: 34 with and 20 without behavioral problems | Cohort: referred to the Royal Children‘s Hospital for suspected hyperactivity and parents reported behavior changes with diet  Selection: unclear  Recruitment: unclear  Participation: unclear  Cross-over: yes  Randomized: yes  Blinded: double  Placebo: yes  Adequate placebo: NA | Tartrazine  Daily dose: 1, 2, 5, 10, 20, 50 mg | On elimination diet: yes  Regimen: each dose given one day at random days over a 21 day period with placebo given at least 2-3 consecutive days in between  Placebo: lactose  Vehicle: colorless capsule  Washout: none, on placebo for the days in between the challenge  Infractions: NA | Irritability, sleep disturbance, restlessness, aggression, attention span | Methods: Behavioral Rating Inventory (BRI) (study specific, single score based on 30 item Likert scales for irritability, sleep disturbance, restlessness aggressiveness, and attention span) and Conners 10-item APTQ – both completed by the parents  Validated: no (the BRI doesn’t seem to be validated)  Timing: daily | **Elimination diet:** NA  **Challenge:**  Parent: 24 “reactors” identified but this is not well defined (see notes). 2 of 20 controls were reactors. Greater behavioral scores following all doses (p<0.05) with apparent dose-response relationship in reactors but not consistently in non-reactors. Average effect sizes in reactors appear >20% in figures  Teacher: NA  Other: NA | Timing: NA  Age effect: not seen  Order effect: NA  Individual results: yes, but few details given and results unclear  Replication: not done | Magnitude (>20%): yes  Statistical significance: yes  Dose-response: yes  Subgroup only: yes, reactors  Funder: Royal Children’s Hospital Research Foundation  Reported conflicts: no information  Full results: full Conners results not given, and “consistent variations” not well defined | - 16 girls and 38 boys - Reactors: “consistent variations in behavior for at least 5 of 6 dose challenges” |
| Rowe, 1988  Location: Australia  Hyperactive: mixed  Responders: yes  Ages: 3-15  N: 8 | Cohort: referral for suspected hyperactivity  Selection: unclear  Recruitment: 9/14 = 64%  Participation: 8/9 = 89%  Cross-over: yes  Randomized: unclear  Blinded: double  Placebo: yes  Adequate placebo: NA | Tartrazine and carmoisine  Daily dose: 50 mg (1.25-2 mg/kg per day in the two reactors) | On elimination diet: yes  Regimen: either placebo, carmoisine or tartrazine given once per day for 126 days. Placebo lead-in periods of 3, 4 or 5 weeks. Carmoisine and tartrazine were each tested for 1 week on two separate occasions (i.e. a total of 4 weeks of dye administration)  Placebo: lactose  Vehicle: capsules  Washout: none, placebo given in between challenges  Infractions: NA | Over-activity, restlessness,  impulsiveness, distractibility, low frustration tolerance, overt aggression, short attention span and sleep disturbance | Methods: daily behavior checklists by parents  Validated: no  Timing: daily | **Elimination diet:** NA  **Challenge:**  Parents: two of eight children demonstrated “significant responses” to both food colorings (e.g. irritability, short attention span) (p<0.05). Effect sizes in these individuals appear >20% based on figures  Teacher: ratings were attempted but incomplete  Other: NA | Timing: 2 hours to 3-4 days in one responder, start time of effects not given in the other (but based on figure seems within one day) but effect lasted 3.5 weeks after last coloring dose  Age effect: responders were 7 and 9 years old  Order effect: NA  Individual results: yes, see results  Replication: not done | Magnitude (>20%): yes  Statistical significance: yes  Dose-response: NA  Subgroup only: no  Funder: no information  Reported conflicts: no information  Full results: no, teacher responses incomplete, group means not given | - 6 boys and 2 girls - Only Phase II results reported here (Phase 1 was used to identify responders) - Both responders were atopic - One reactor did not have “inattention” as a feature (which is the focus of the Conners questionnaire) - All reactors to the dye challenge were atopic |
| Sarantinos et al., 1990  Location: Canada  Hyperactive: yes (ADHD)  Responders: mixed  Ages: 4-14  N: 13 | Cohort: previously diagnosed with ADHD  Selection: unclear  Recruitment: unclear  Participation: unclear  Cross-over: yes  Randomized: yes  Blinded: double  Placebo: yes  Adequate placebo: the mother of one responder correctly identified coloring vs. placebo in 25 of 28 occasions | Tartrazine and sunset yellow  Daily dose: 60 mg | On elimination diet: yes  Regimen: one group received six challenges of tartrazine (10 mg per challenge), and one group received three daily challenges of tartrazine (10 mg per challenge) and three daily challenges of sunset yellow (10 mg per challenge)  Placebo: orange juice with the challenge  Vehicle: orange juice  Washout: unclear, children may have received the placebo between active challenges  Infractions: NA | Hyperactivity, others | Methods: Conners Abbreviated Parents Scale and Behavioral Rating Inventory, both by parents  Validated: yes  Timing: daily | **Elimination diet:** NA  **Challenge:**  Parent: two children showed a significant change in behavior with both dyes on both scales (p<0.05). Few details provided, group means or effect sizes in individuals not given  Teacher: NA  Other: NA | Timing: NA  Age effect: NA  Order effect: NA  Individual results: yes, see main results  Replication: not done | Magnitude (>20%): unclear  Statistical significance: yes  Dose-response: NA  Subgroup only: no  Funder: no information  Reported conflicts: no information  Full results: no, group means or detailed individual results not given | - 1 girl and 12 boys - In 4 of the children, parents were uncertain of improvement on synthetic color free diet - Both responders were atopic |
| Spring et al., 1981  Location: US  Hyperactive: likely  Responders: yes  Ages: 8-13  N: 6 | Cohort: previous responders  Selection: unclear  Recruitment: unclear  Participation: 6/8 = 75%  Cross-over: yes  Randomized: yes  Blinded: double  Placebo: yes  Adequate placebo: active challenge could not be distinguished from placebo in pilot testing (few details given) | Feingold diet  Red 40, yellow 5, yellow 6, red 3, blue 1, blue 2, orange B, green 3  Daily dose: 26 mg | On elimination diet: yes  Regimen: cookies with (active) or without (placebo) synthetic food colors eaten three consecutive days per week for two weeks each, with elimination diet in between  Placebo: cookies without synthetic dyes  Vehicle: cookies  Washout: 4 days  Infractions: low rate of infractions in the responding subject, greater infractions in 3 others | Hyperactivity, overall behavior | Methods: abbreviated study specific hyperactivity rating scale by parents, similar to the Conners abbreviated scale. Mothers and teachers asked to guess whether child had been given active challenge or placebo  Validated: yes  Timing: 3 days per week | **Elimination diet:**  Parent: hyperactivity ratings all decreased while on the elimination diet (improvement of 49%, p-value not given)  Teacher: NA  Other: NA  **Challenge:**  Parent: guesses were fairly accurate in one subject (“Subject E”) and moderately accurate in another subject. In Subject E, correlations seen between challenge and hyperactivity scores (p< 0.05). Inaccurate in all others  Teacher: guesses were accurate in Subject E. Inaccurate in all others  Other: NA | Timing: NA  Age effect: NA  Order effect: unclear  Individual results: yes, positive results mostly confined to one individual  Replication: results in Subject E could not be replicated | Magnitude (>20%): no  Statistical significance: no (initial result not replicated)  Dose-response: NA  Subgroup only: no  Funder: no information  Reported conflicts: no information  Full results: yes | - 6 Caucasian boys - Medications were discontinued |
| Swanson and Kinsbourne, 1980a  Location: Canada  Hyperactive: mixed  Responders: no  Ages: 5-12  N: 40 | Cohort: referrals with hyperactive symptoms  Selection: unclear  Recruitment: unclear  Participation: unclear  Cross-over: yes  Randomized: unclear  Blinded: unclear  Placebo: yes  Adequate placebo: NA | A blend of nine food dyes, in proportion to use in the US  Daily dose: 100 (n=20) and 150 mg (n=20) | On elimination diet: yes  Regimen: Feingold diet for 3 days then challenged with food coloring or placebo once per day on days 4 and 5  Placebo: capsule with sugar  Vehicle: capsules  Washout: none  Infractions: monitored | Learning task; hyperactivity | Methods: paired associate learning test; Conners scale (unclear who filled this out)  Validated: yes  Timing: four times per day | **Elimination diet:** NA  **Challenge:**  Parents: NA  Teachers: NA  Other: increase in errors associated with food dyes (p<0.05). Similar effects with 100 or 150 mg doses. Effects only seen in those who previously responded to methylphenidate. No associations seen on the Conners scale  Large placebo effect seen in the non-hyperactive group | Timing: effects took ½ hour to become evident peaked at 1.5 hours after dosing, and lasted at least 3.5 hours.  Age effect: NA  Order effect: NA  Individual results: not given  Replication: not done | Magnitude (>20%): unclear  Statistical significance: yes  Dose-response: not seen  Subgroup only: yes, previously responded to methylphenidate  Funder: no information  Reported conflicts: no information  Full results: yes | - 36 boys and 4 girls - Children were hospitalized during the study - Included 20 children who showed a favorable response to stimulant medication (methylphenidate) (“hyperactive”) and 20 who did not - Medications were stopped during the study - Specific dyes and percentages given in the article |
| Swanson and Kinsbourne, 1980b  Location: Canada  Hyperactive: yes  Responders: no  Ages: unclear  N: 8 | Cohort: unclear  Selection: unclear  Recruitment: unclear  Participation: unclear  Cross-over: yes  Randomized: unclear  Blinded: double  Placebo: yes  Adequate placebo: unclear | “color blend”, not described  Daily dose: 26 mg | On elimination diet: yes  Regimen: Feingold diet for 2 days then challenged with placebo on day 3, and food colorings on days 4 and 5  Placebo: cookie without food dyes  Vehicle: cookie  Washout: none  Infractions: monitored intakes | Learning task; hyperactivity | Methods: paired associate learning test; Conners scale (teacher and learning test administrator)  Validated: yes  Timing: six times per day | **Elimination diet:** NA  **Challenge:**  Parents: NA  Teachers: NA  Other: increase in performance with active ingredient vs. placebo (opposite of expectation). Conners scores not given. | Timing: no effects  Age effect: NA  Order effect: NA  Individual results: not given  Replication: not done | Magnitude (>20%): no  Statistical significance: no  Dose-response: NA  Subgroup only: NA  Funder: no information  Reported conflicts: no information  Full results: no Conners scores | - Children were hospitalized during the study - Medications were stopped - Specific dyes and percentages not given - Higher doses assessed in Swanson and Kinsbourne, 1980a and protocols somewhat different |
| Thorley, 1984  Location: UK  Hyperactive: unclear  Responders: no  Ages: 12 (mean)  N: 10 | Cohort: in residence boarders at a school for developmentally disabled  Selection: all  Recruitment: 100%  Participation: 100%  Cross-over: yes  Randomized: yes  Blinded: double  Placebo: yes  Adequate placebo: may have been assessed but no details given | 16 of the most commonly used dyes  Daily dose: 91.8 mg | On elimination diet: yes  Regimen: 14 day trial with challenge once per day for 2 consecutive days, with placebo on other days  Placebo: cocoa drink without challenge  Vehicle: cocoa drink  Washout: likely adequate, it appears that only tests done on the placebo days prior to challenge were used as the comparison, 14 days on elimination diet prior to study  Infractions: none | Hyperactivity, others | Method: Conners scale reported by teachers and care staff; study specific scale of the “five most problematic behaviors; other tests (Porteus Mazes, paired associate learning test, actometers)  Validated: yes  Timing: testing -2 hours after ingestion, behavioral observations throughout the day | **Elimination diet:** NA  **Challenge:**  Parent: scores improved on challenge days (opposite of expectation)  Teacher: scores improved on challenge days (opposite of expectation)  Other: Porteus mazes, paired-associate learning test and actometer readings and scores were worse with the challenge (5-30%) but results not statistically significant | Timing: NA  Age effect: NA  Order effect: NA  Individual results: not given  Replication: not done | Magnitude (>20%): yes  Statistical significance: no  Dose-response: NA  Subgroup only: no  Funder: no information  Reported conflicts: no information  Full results: yes | - 8 boys and 2 girls - Specific dyes and percentages provided in the article |
| Weiss et al., 1980  Location: US  Hyperactive: no  Responders: yes  Ages: 2-7  N: 22 | Cohort: members of Kaiser  Selection: unclear  Recruitment: unclear  Participation: unclear  Cross-over: yes  Randomized: yes  Blinded: double  Placebo: yes  Adequate placebo: NA | 7 food colors plus cranberry coloring  Daily dose: 35 mg | On elimination diet: yes  Regimen: challenge given 8 days randomly distributed over 8 week period  Placebo: soft drink with caramel and cranberry coloring  Vehicle: soft drink  Washout: placebo given on all non-active challenge days  Infractions: NA | Adverse behaviors | Method: parent ratings using 7 bad and 3 good behaviors selected by each parent; Conners - parents  Validated: main scale not validated  Timing: parent ratings two times per day, each day, once within 3.5 hours of dosing and once “at a later time”, and an overall global estimate at the end of each day; Conners (likely daily) | **Elimination diet:** NA  **Challenge:**  Parent: challenge associated with increase in adverse behaviors seen in 2 of 22 children (p<0.05 in both subjects). Effect size appears >20% based on figures  Teacher: NA  Other: NA | Timing: NA  Age effect: NA  Order effect: NA  Individual results: yes, see main results  Replication: not done | Magnitude (>20%): yes  Statistical significance: yes  Dose-response: NA  Subgroup only: no  Funder: NIH, US FDA, Kaiser, US Department of Energy  Reported conflicts: no information  Full results: no, group means not given and Conners scores only given in one child | - 15 boys and 7 girls - Doses of each dye given in their Table 1 |
| Williams et al., 1978  Location: Canada  Hyperactive: yes  Responders: no  Ages: 6-12  N: 28 | Cohort: hyperactive children  Selection: unclear  Recruitment: 29/38= 76%  Participation: 26/29= 90%  Cross-over: yes  Randomized: yes  Blinded: double  Placebo: yes  Adequate placebo: NA | Red dyes 2, 3, and 4; blue dyes 1  and 2; yellow dyes 5 and 6; green dye 3; and orange dye B  Daily dose: ½ dietary intake in the US per cookie | On elimination diet: yes  Regimen: two cookies 4 consecutive days per week, one week for the active challenge and one week for the placebo  Placebo: cookie without added dyes  Vehicle: chocolate cookie  Washout: 3 days  Infractions: 7 children had infractions, all ≤2 per week, unrelated to behavioral scores | Hyperactivity | Method: Conners scales 11, 40, 96 items by parents and teachers  Validated: yes  Timing: 11-item: 4 days per week by parents and 2 days per week by teachers; 40 item: once per week parents and teachers; 96 item: at beginning and end of trial by parents | **Elimination:** NA  **Challenge:**  Parent: average ratings higher for challenge vs. placebo but primarily in those also receiving medications (with effect sizes appearing >20% in figures), and differences are not statistically significant  Teacher: statistically significant findings in those not receiving medications, effect size appears >20% in figures  Other: NA  Effects expressed only as f values | Timing: NA  Age effect: NA  Order effect: NA  Individual results: 3 and 5 children were identified who had a 33% change in scores with challenge vs. placebo in parent and teacher scores, respectively, but no overlap  Replication: may have been done but no results given | Magnitude (>20%): yes  Statistical significance: yes  Dose-response: NA  Subgroup only: yes, stronger effects in those not receiving medications  Funder: PSI Foundation  Reported conflicts: no information  Full results: no, mostly given in figure form and f-scores | - 2 girls and 26 boys - Medications were also tested with food dyes in a 2 x 2 factorial design - Correlations between teacher and parent ratings were 0.2-0.4 - Actual doses not given in the article |
| Wilson and Scott, 1989  Location: UK  Hyperactive: no  Responders: yes  Ages: 2-14  N: 19 | Cohort: unclear  Selection: unclear  Recruitment: unclear  Participation: 19/29= 66%  Cross-over: yes  Randomized: yes  Blinded: double  Placebo: yes  Adequate placebo: NA | Sunset yellow and tartrazine  Daily dose: 8.5 mg | On elimination diet: yes  Regimen: one bottle of challenge per day for 12 days, and one bottle of placebo per day for 12 days, each followed by a 2 day washout period  Placebo: Lucozade drink with synthetic beta-carotene  Vehicle: Lucozade drink  Washout: 2 days, if symptoms not returned to baseline then 1 week  Infractions: NA | Any symptom, symptom scores | Method: parents assessment whether behaviors worsened (appears very non-specific)  Validated: no  Timing: unclear | **Elimination diet:** NA  **Challenge:**  Parent: 3 of 19 children had symptoms associated with food coloring with repeated testing but all were allergic (urticarial, eczema, or asthma). No behavioral problems linked to food colorings  Teacher: NA  Other: NA | Timing: NA  Age effect: NA  Order effect: NA  Individual results: yes, see results  Replication: yes | Magnitude (>20%): no  Statistical significance: no  Dose-response: NA  Subgroup only: no  Funder: Asthma Research Council, Beecham Group  Reported conflicts: no information  Full results: no, group means and actual behavioral scores not given | - Prior symptoms included behavioral disturbances, allergic symptoms, GI upset - Trial was repeated if associated symptoms identified - Sodium benzoate also tested |

Abbreviations: ADHD, attention deficit hyperactivity disorder; FDA, US Food and Drug Administration; KP, Kaiser Permanente; N, number of participants; NA, not assessed; NIH, US National Institutes of Health; P-TQ, Conners Parent-Teacher Questionnaire; WISC, Wechsler Intelligence Scale for Children

Rows are sorted by study first author.

Numbers in parentheses following relative risk estimates or means are 95% confidence intervals unless otherwise noted. Ages are in years unless otherwise noted

##### Table A.3 Clinical trials of synthetic food dyes and neurobehavioral outcomes in children: coding

| Study | Publication_year | Location | Hyperactive | Prior_responders | Ages | N | Boys/Girls | Elimination_tested | Challenge_tested | Dose | Other_agent | Washout_method | Washout_period | Outcome_hyperactive | Outcome_other | Timing_outcome | Results_elimination | Parent_grp_chall | Teacher_grp_chall | Other_grp_chall | Parent_Individ_chall | Teacher_individ_chall | Other_individ_chall | Timing_effect | Age_effect | Dose_response | Subgroup_only | Random_sample | Dropouts_low | Crossover | Random_crossover | Double_blinded | Exposure_defined | Food_dyes_only | Multiple_doses | High_dose | Placebo_tested | Washout_adequate | On_elim_diet | Outcome_relevant | Outcome_validated | Individ_results_given | Replication_done | Infractions_low | No_order_effect | No_conflicts | Full_results |
| --- | --- | --- | --- | --- | --- | --- | --- | --- | --- | --- | --- | --- | --- | --- | --- | --- | --- | --- | --- | --- | --- | --- | --- | --- | --- | --- | --- | --- | --- | --- | --- | --- | --- | --- | --- | --- | --- | --- | --- | --- | --- | --- | --- | --- | --- | --- | --- |
| Adam | 1981 | US | 1 | 1 | 4-11 | 18 | 15/3 | 0 | M | 26.3 | 0 | E | U | U | M | H | N | 0 | N | 0 | N | N | N | N | 0 | N | 0 | 0 | 0 | 1 | 1 | 1 | 1 | 1 | 0 | 0 | 0 | 0 | 1 | 0 | 0 | 0 | 0 | 1 | 1 | 0 | 0 |
| Batem | 2004 | UK | 2 | 0 | 3 | 277 | 151/  126 | 1 | M | 20 | P | E | 7 | 1 | M | D | 2 | 2 | N | 0 | N | N | N | N | N | N | 0 | 1 | 1 | 1 | 1 | 1 | 1 | 0 | 0 | 0 | 1 | 1 | 1 | 1 | 1 | 0 | 0 | 1 | 1 | 0 | 1 |
| Con76 | 1976 | US | 1 | 0 | 6-12 | 15 | U | 1 | 0 | - | 0 | 0 | 0 | 1 | 0 | W | 2 | N | N | N | N | N | N | N | N | N | 0 | 0 | 0 | 1 | 1 | 0 | 1 | 0 | - | - | 0 | 0 | - | 1 | 1 | 0 | 0 | 1 | 0 | 0 | 1 |
| Con80 | 1980 | US | 1 | 1 | 5-10 | 9 | U | 0 | M | 15 | 0 | E | 7 | 1 | M | H | N | N | N | 0 | N | N | N | N | N | N | 0 | 0 | 0 | 1 | 1 | 1 | 0 | 1 | 0 | 0 | 0 | 1 | 1 | 1 | U | 0 | 0 | 1 | 1 | 0 | 1 |
| David | 1987 | UK | 0 | 1 | 1-12 | 24 | 19/5 | 0 | T | 250 | 0 | E | 30 | U | M | U | N | 0 | N | 0 | 0 | N | 0 | N | N | 0 | 0 | 1 | 1 | 1 | 0 | 1 | 1 | 1 | 1 | 1 | 0 | 1 | 1 | 0 | 0 | 1 | 0 | 1 | 0 | 0 | 1 |
| Goy | 1978 | US | 1 | 1 | 4-12 | 16 | U | 1 | M | 26 | 0 | 0 | 0 | 1 | M | H | 1 | 2 | 0 | 0 | N | N | 1 | H | Y | N | 0 | 0 | 0 | 1 | 0 | 1 | 0 | 1 | 0 | 0 | 0 | 0 | 1 | 1 | 1 | 1 | 1 | 0 | 1 | 0 | 0 |
| Har78a | 1978 | US | 2 | 0 | 3-13 | 80 | 80/0 | 1 | 0 | - | 0 | U | U | 1 | M | S | 2 | N | N | N | N | N | N | N | Y | N | 0 | 0 | 0 | 1 | 1 | 1 | 1 | 0 | - | - | 1 | 0 | - | 1 | 1 | 1 | 0 | 1 | 1 | 0 | 0 |
| Har78b | 1978 | US | 2 | 1 | 3-12 | 18 | 18/0 | 0 | M | 54 | 0 | 0 | 0 | 1 | M | S | N | 0 | 0 | 0 | 1 | U | 1 | N | N | N | 0 | 0 | 0 | 1 | 0 | 1 | 0 | 1 | 0 | 1 | 1 | 0 | 1 | 1 | 1 | 1 | 0 | 1 | 0 | 0 | 1 |
| L&H78 | 1978 | AU | 1 | 1 | 5 | 8 | 7/1 | 0 | T | 4 | 0 | U | U | 1 | 0 | U | N | 0 | N | N | N | N | N | N | N | N | 0 | 0 | 1 | 1 | 1 | 0 | 1 | 1 | 0 | 0 | 1 | 0 | 1 | 1 | 1 | 0 | 0 | 0 | 0 | 0 | 0 |
| Levy78 | 1978 | AU | 1 | 0 | 4-8 | 22 | 19/3 | 1 | T | 5 | 0 | 0 | 0 | 1 | M | G | 2 | 2 | 0 | 0 | N | N | N | N | N | N | H | 0 | 0 | 1 | 0 | 1 | 1 | 1 | 0 | 0 | 0 | 0 | 1 | 1 | 1 | 0 | 0 | 1 | 0 | 0 | 0 |
| Lok | 2013 | AS | 0 | 0 | 8-9 | 130 | 70/60 | 0 | M | 62.4 | 0 | E | 7 | 1 | M | W | N | 0 | 0 | N | N | N | N | N | N | N | 0 | 1 | 1 | 1 | 1 | 1 | 1 | 1 | 0 | 1 | 0 | 1 | 1 | 1 | 1 | 0 | 0 | 1 | 0 | 1 | 1 |
| M&G81 | 1981 | US | 2 | 1 | 4-13 | 11 | 6/5 | 0 | M | 78 | 0 | E | 7 | 1 | M | H | N | 0 | 0 | 0 | U | U | U | N | 0 | N | 0 | 0 | 1 | 1 | 1 | 1 | 0 | 1 | 0 | 1 | 1 | 1 | 1 | 1 | 1 | 0 | 0 | 1 | 1 | 0 | 1 |
| M&GK | 1978 | US | 1 | 1 | 10 | 1 | 1/0 | 0 | M | U | 0 | E | 5 | 1 | 0 | W | N | N | N | N | 2 | 0 | N | N | N | N | 0 | 0 | 1 | 1 | 1 | 1 | 0 | 1 | 0 | 0 | 0 | 1 | 1 | 1 | 1 | 1 | 1 | 0 | 0 | 0 | 1 |
| McCa | 2007 | UK | 0 | 0 | 3-9 | 297 | 154/  143 | 0 | M | 64 | P | U | U | 1 | M | W | N | N | N | 2 | N | N | N | N | Y | N | 0 | 0 | 1 | 1 | 1 | 1 | 1 | 0 | 0 | 1 | 1 | 0 | 1 | 1 | 1 | 0 | 0 | 1 | 0 | 1 | 1 |
| Pollo | 1990 | UK | 2 | 1 | 2-15 | 19 | U | 0 | M | 125 | 0 | E | 7 | 1 | M | D | N | 2 | N | N | 1 | N | N | 0 | N | N | 0 | 0 | 0 | 1 | 1 | 1 | 1 | 1 | 0 | 1 | 0 | 1 | 1 | 1 | 1 | 1 | 0 | 0 | 1 | 0 | 1 |
| Rapp | 1978 | US | 1 | 0 | 5-16 | 24 | 18/6 | 0 | M | U | 0 | 0 | 0 | 1 | M | H | N | N | N | 1 | N | N | 1 | N | N | N | 0 | 0 | 0 | 1 | 1 | 1 | 0 | 1 | 0 | 0 | 0 | 0 | 0 | 1 | 0 | 1 | 0 | 1 | 0 | 0 | 0 |
| Rose | 1978 | US | 1 | 1 | 8 | 2 | 0/2 | 0 | T | 1.2 | 0 | U | U | 1 | M | D | N | 2 | N | 2 | 2 | N | 2 | N | N | N | 0 | 0 | 0 | 1 | 0 | 1 | 1 | 1 | 0 | 0 | 1 | 0 | 1 | 1 | 1 | 1 | 0 | 1 | 0 | 0 | 0 |
| Rowa94 | 1994 | AU | 2 | 2 | 2-14 | 54 | 38/16 | 0 | T | 50 | 0 | 0 | 0 | 1 | M | D | N | 2 | N | N | 1 | N | N | N | 0 | 1 | 1 | 0 | 0 | 1 | 1 | 1 | 1 | 1 | 1 | 1 | 0 | 0 | 1 | 1 | 0 | 1 | 0 | 0 | 0 | 0 | 0 |
| Rowb88 | 1988 | AU | 2 | 1 | 3-15 | 8 | 6/2 | 0 | M | 50 | 0 | 0 | 0 | 1 | M | D | N | N | N | N | 2 | N | N | H | U | N | 0 | 0 | 1 | 1 | 0 | 1 | 1 | 1 | 0 | 1 | 0 | 0 | 1 | 1 | 0 | 1 | 0 | 0 | 0 | 0 | 0 |
| Sara | 1990 | CA | 1 | 2 | 4-14 | 13 | 12/1 | 0 | T | 60 | 0 | 0 | 0 | 1 | M | D | N | N | N | N | 2 | N | N | N | N | N | 0 | 0 | 0 | 1 | 1 | 1 | 1 | 1 | 0 | 1 | 1 | 0 | 1 | 1 | 1 | 1 | 0 | 0 | 0 | 0 | 0 |
| Spring | 1981 | US | 1 | 1 | 8-13 | 6 | 6/0 | 1 | M | 26 | 0 | E | 4 | 1 | M | S | 1 | N | N | N | 0 | N | N | N | N | N | 0 | 0 | 1 | 1 | 1 | 1 | 1 | 1 | 0 | 0 | 1 | 1 | 1 | 1 | 1 | 1 | 1 | 1 | 0 | 0 | 1 |
| SKa | 1980 | CA | 2 | 0 | 5-12 | 40 | 36/4 | 0 | M | 150 | 0 | 0 | 0 | 1 | M | H | N | N | N | 2 | N | N | N | H | N | 0 | M | 0 | 0 | 1 | 0 | 0 | 1 | 1 | 1 | 1 | 0 | 0 | 1 | 1 | 1 | 0 | 0 | 1 | 0 | 0 | 1 |
| SKb | 1980 | CA | 1 | 0 | U | 8 | U | 0 | 1 | 26 | 0 | 0 | 0 | 1 | M | H | N | N | N | 0 | N | N | N | 0 | N | N | 0 | 0 | 0 | 1 | 0 | 1 | 0 | 1 | 0 | 0 | 0 | 0 | 1 | 1 | 1 | 0 | 0 | 1 | 0 | 0 | 0 |
| Thorl | 1984 | UK | U | 0 | 12 | 10 | 8/2 | 0 | M | 91.8 | 0 | E | 14 | 1 | M | H | N | 0 | 0 | 1 | N | N | N | N | N | N | 0 | 1 | 1 | 1 | 1 | 1 | 1 | 1 | 0 | 1 | 0 | 1 | 1 | 1 | 1 | 0 | 0 | 1 | 0 | 0 | 1 |
| Weiss | 1980 | US | 0 | 1 | 2-7 | 22 | 15/7 | 0 | M | 35 | Y | 0 | 0 | 1 | M | H | N | N | N | N | 2 | N | N | N | N | N | 0 | 0 | 0 | 1 | 1 | 1 | 1 | 0 | 0 | 0 | 0 | 0 | 1 | 1 | 0 | 1 | 0 | 0 | 0 | 0 | 0 |
| Willia | 1978 | CA | 1 | 0 | 6-12 | 28 | 26/2 | 0 | M | U | 0 | E | 3 | 1 | M | S | N | 1 | 2 | N | 1 | 1 | N | N | N | N | M | 0 | 1 | 1 | 1 | 1 | 0 | 1 | 0 | 0 | 0 | 1 | 1 | 1 | 1 | 1 | 0 | 1 | 0 | 0 | 0 |
| Wils | 1989 | UK | 0 | 1 | 2-14 | 19 | 11/8 | 0 | M | 8.5 | 0 | E | 7 | U | M | U | N | N | N | N | 0 | N | N | N | N | N | 0 | 0 | 0 | 1 | 1 | 1 | 1 | 1 | 0 | 0 | 0 | 1 | 1 | 0 | 0 | 1 | 1 | 0 | 0 | 0 | 0 |

Variable names are formatted for analysis in SAS

Bolded variable names are those used for quality scoring

Coding dictionary is provided in the following table

Other coding notes:

1. David et al., 1987: unclear if a placebo was used. The comparison appears to be the time before the challenge – a time which all children were on an elimination diet. As such, placebo_used labeled as “0”
2. Mattes and Gittleman, 1981 and McCann et al., 2007: multiple dose levels used, but results not provided for each dose level. As such, multiple_doses labeled as “0”
3. Rose, 1978: study only included two subjects but results were the same in each so “group” results were recorded the same as “individual” results
4. Sarantinos et al., 1990: one group received tartrazine only and one group received a combination of tartrazine and sunset yellow
5. Spring et al., 1981: findings were recorded as negative since the initial findings were not confirmed in the replication study. Teacher ratings were not done in the replication study so were recorded as “N”
6. Thorley, 1984: we estimated 6 days on average for the washout period (14 day trial, received challenge in randomly selected 2 consecutive days during this time)

| Table A.4. Coding dictionary | | | | |
| --- | --- | --- | --- | --- |
| **Variable name** | **Category** | **Definition** | **Codes** | **Notes** |
| Study | Characteristic | Abbreviated study name |  |  |
| Publication_year | Characteristic | Publication year | Year | Few articles give the actual dates the study was done |
| Location | Characteristic | Where was the study done? | AS=Asia, AU=Australia, CA=Canada, O=other, U=US, UK=United Kingdom | If the location was not specifically provided then the institution of the first author used here |
| Hyperactive | Characteristic | Did the study only included participants who were diagnosed as hyperactive, had hyperactive symptoms, or a related condition? | 1=yes, 0=no, 2=mixed, U=unclear | Includes any condition related to hyperactivity (e.g. ADHD) |
| Prior_responders | Characteristic | Did the study only include participants who had previously reported improvements on Feingold or similar diet? | 1=yes, 0=no, 2=mixed, U=unclear |  |
| Ages | Characteristic | Age range of the participants | Range | A single number is given if only the average age is provided in the article |
| N | Characteristic | Number of participants | Number |  |
| Boys/Girls | Characteristic | Number of boys/number of girls | Number; U=unclear or unknown |  |
| Elimination_tested | Characteristic | Was an elimination diet tested? | 1=yes, 0=not tested, U=unclear | If yes, this was almost always a version of the Feingold or KP diet |
| Challenge_tested | Characteristic | If there was a challenge with artificial food dyes, what were the dyes? | M=multiple dyes, 0=not tested, T=tartrazine |  |
| Dose | Characteristic | What was the total daily dose of all food dyes tested? | In mg, “-“ specific dyes not tested, U=unknown or unclear | If multiple doses, the highest dose is given |
| Other_agent | Characteristic | Was another agent tested in addition to food dyes and if so what? | 0=none, P=preservative (e.g benzoic acid), Y=yes, other |  |
| Washout_method | Characteristic | What was given to the participant between the placebo and active challenges? | 0=no washout, E=elimination diet, P=placebo only, U=unclear |  |
| Washout_period | Characteristic | If the placebo was given after the challenge, how many days after the challenge was it given? | In days, U=unclear, 30=if likely >30 days, 0=no washout |  |
| Outcome_hyperactive | Characteristic | Was hyperactivity or a related outcome tested? | 1=yes, 0=no, U=unclear |  |
| Outcome_other | Characteristic | If an outcome other than hyperactivity was tested, what was it? | 0= none, M=multiple |  |
| Timing_outcome | Characteristic | When was the outcome assessed relative to when the challenge was given? | H=about hourly or at least 2-3 times per day, D=daily, S=several times per week, W=weekly, G=greater than weekly, O=other, U=unclear |  |
| Results_elimination | Results | If an elimination diet was tested, what were the results? | 0=no association, 1=≥20% effect size but not statistically significant (or statistical significance not given), 2=statistically significant, U=unclear, N=not tested |  |
| Parent_grp_challenge | Results | Parent behavioral rating results in the group as a whole for an active challenge vs. placebo | Same as above | Results only for a challenge (where an artificial food dye is given), not for an elimination diet trial |
| Teacher_grp_challenge | Results | Teacher behavioral rating results in the group as a whole for an active challenge vs. placebo | Same as above | Same as above |
| Other_grp_challenge | Results | Other results in the group as a whole for an active challenge vs. placebo | Same as above | Same as above |
| Parent_individ_chall | Results | Did any individual show a challenge effect on parent ratings? | Same as above | Same as above |
| Teacher_individ_chall | Results | Did any individual show a challenge effect on teacher ratings? | Same as above | Same as above |
| Other_individ_challenge | Results | Did any individual show a challenge effect on any other test? | Same as above | Same as above |
| Timing_effect | Results | Was data on latency presented, and if so what were the results? | H=effects found in the first 1-2 hours of the challenge, 0=similar results at different time points, N=not assessed | Time from challenge to when effects began, did they compare shorter to longer latencies? |
| Age_effect | Results | Did certain age groups show a greater challenge effect? | Y=young (most <5 years old), Ol=older, 0=tested but age effect not seen, N=not assessed, U=unclear |  |
| Dose_response | Results | Was a dose-response relationship found? | N=not assessed, 0=tested but not found, 1=relationship found, U=unclear |  |
| Subgroup_only | Results | Was the association confined to a subgroup of the whole study population? | H=if effect only seen in hyperactive group, M=medication-related, 1=another specific subgroup, 0=no specific subgroup identified |  |
| Random_sample | Quality | Were the participants randomly selected or was the entire cohort included? | 1=yes, 0=no or unclear |  |
| Dropouts_low | Quality | Did ≤30% of participants who started the study drop out? | 1=yes, 0=no or unclear |  |
| Crossover | Quality | Did each participant receive both the active agent and the placebo? | 1=yes, 0=no or unclear |  |
| Random_crossover | Quality | Was the order of placebo vs. active ingredient assigned randomly? | 1=yes, 0=no, unclear, or not a cross-over design |  |
| Double_blinded | Quality | Were both the observer and the participant blinded to the exposure given? | 1=yes, 0=no or unclear |  |
| Exposure_defined | Quality | Were the agents tested and their exact doses provided? | 1=yes, 0=no or unclear |  |
| Food_dyes_only | Quality | Do results represent those of only artificial food dyes? | 1=yes, 0=no or unclear | “0” if food dyes were combined with another agent |
| Multiple_doses | Quality | Were multiple dose levels tested? | 1=yes, 0=no or unclear | Was dose-response assessed? |
| High_dose | Quality | Was a high dose tested? | 1=dose ≥50 mg tested, 0=lower or unknown dose |  |
| Placebo_tested | Quality | Was it shown that the placebo and the active challenge could not be distinguished? | 1=yes, 0=no or unclear |  |
| Washout_adequate | Quality | Was there a washout period of at least 2 days? | 1=yes, 0=no, unclear, or not reported |  |
| On_elim_diet | Quality | If a challenge study, were the subjects on an elimination diet during the challenge | 1=yes, 0=no, unclear, or not reported |  |
| Outcome_relevant | Quality | Was an outcome similar or relevant to hyperactivity assessed? | 1=yes, 0=no or unclear |  |
| Outcome_validated | Quality | Was the method used to assess the outcome validated? | 1=yes, 0=no, unclear, or not reported |  |
| Individ_results_given | Quality | Were the results in each child given (i.e. not just group means)? | 1=yes, 0=no or unclear |  |
| Replication | Quality | Were positive findings replicated? | 1=yes, 0=no, unclear, or no positive findings |  |
| Infractions | Quality | Were dietary infractions low (<2 per week)? | 1=yes or monitored dosing, 0=no, unclear, or not reported |  |
| No_order_effect | Quality | Results were found not to be dependent on order the active challenge or placebo were given | 1=yes, 0=no or unclear |  |
| No_conflicts | Quality | Were potential conflicts reported? | 1=yes, 0=no, unknown, or unclear | Includes funding source and other potential conflicts |
| Full_results | Quality | Were full results reported? | 1=yes, 0=no or unclear | Includes group means, variance, individual results, and probability of chance |

**Animal Toxicity Summary Table**

##### Table A.5 Individual dyes. Developmental and adolescent/adult studies.

Results columns present statistically significant differences between a dose group and control group reported by authors. Statistically significant dose trend tests reported by the authors are also presented. Arrow (↑/↓) indicates direction of difference from control group. For additional variable measurements, statistically significant differences with dye-treatment exposures are presented. GD=gestational day; PND=postnatal day.

| **Study Information** | **Experimental Design** | **Dye Information** | **Exposure** | **Outcome Assessed** | **Behavioral Results** | **Brain Measurements Results** |
| --- | --- | --- | --- | --- | --- | --- |
| Reference: (Sobotka et al. 1977)  **Institution**: Division of Toxicology, US FDA  **Funding Source:** US FDA  **Ethical Statement**: Not provided  **Conflict of Interest**: Not provided | **Species**: Rats, Sprague-Dawley  **Sex**: Male and female  **Group Size:** Behavior:  *Dams*: 4/group *Offspring*: *Behavior:* preweaning development 19-20 males & females/group; postweaning 8-10 males & females  Brain: *Dams*: 4-6 group *Offspring*: 10 males/group  **Exposure Duration:** GD 7 to end of PND 90  **Age at test:** PND 0-90 | **Dye Name**:  Yellow No. 5  **Purity Level:** 93%  **Dye Source:** H. Kohnstamm & Co. | **Route of Administration**: Diet  **Doses**: 0%, 1% and 2% diet  **Control**: 0% Diet | *Dams:*  activity  *Offspring*: Preweaning development (right reflex, neuromotor clinging ability, auditory startle response, placing response and motor activity)  Avoidance learning  Brain assays: (telencephalon, brainstem, cerebellum; weight, protein, cholesterol, DNA) | Preweaning development:  Females: ↑ clinging at 1% and 2% diet, PND 4, 6 and 8 | No dye treatment effects |
| **Reference:** (Vorhees et al. 1983a)  **Institution**: Children’s Hospital Research Foundation  **Funding Source:** US FDA 223-75-2030 (partial)  **Ethical Statement**: Not provided  **Conflict of Interest**: Not provided | **Species**: Rats, Sprague Dawley  **Sex**: Male and female  **Group Size:**  *Dams:* 10-18 /group *Offspring behavior*: 10-18 males, 10-18 females/group *Offspring brain measures*: not stated  Two separate experiments:  Exp. 1: Version 3 test battery + positive control, hydroxyurea  Exp. 2: Version 9 test battery doses without positive control  **Exposure Duration:** 2 weeks premating to PND 90-110  **Age at test:** Preweaning; PND 30-112 | **Dye Name**:  Red No. 3  **Purity Level:** 91%  **Dye Source:** H. Kohnstamm & Co. | **Route of Administration**: Diet  **Doses**: 0%, 0.25%, 0.5% and 1% diet  **Control**: 0% diet | Preweaning development (surface righting, pivoting, cliff avoidance (Exp. 1 only), negative geotaxis, auditory startle, swimming ontogeny, open field, olfactory orientation (Exp.2 only)  Swimming development  Preweaning open-field  Postweaning open-field  Operant discrimination (Exp. 1 only)  Brightness discrimination (Exp. 2 only)  Rotorod  Active avoidance  Water maze (Exp. 2 only)  Passive avoidance  Running wheel activity  Brain measurements  Brain region weights | Swimming development: Exp. 1: ↑ swimming angle development at 1%, 0.25% diet, PND 10  Exp. 2: ↑ swimming angle development at 0.5% and 0.25% diet, PND 10  Exp. 2: ↓ swimming direction at 1% diet, PND 6  Postweaning open-field: Exp.1: ↑ activity at 0.25% and 1% diet, PND 15-17  Exp. 1: ↑ defecation at 0.5%, PND 15-17  Passive avoidance:  Exp. 1 : ↑ entry latency at 0.25% diet, PND 110-112  Running wheel activity: Exp. 1: Females: ↑ activity at 0.25% diet, PND 30-50  Exp. 2: males and females: ↑ activity at 0.5% diet, PND 25-45 | Brain measurements:  Exp. 2: ↑ cerebellar weight at 0.25% and 0.5% diet |
| **Reference:** (Vorhees et al. 1983b)  **Institution**: Cincinnati Children’s Hospital Research Foundation  **Funding Source:** US FDA Project 223-75-2030 (partial)  **Ethical Statement**: Not provided  **Conflict of Interest**: Not provided | **Species**: Rats, Sprague Dawley  **Sex**: Male and female  **Group Size:**  *Dams*: 9-15/group *Offspring behavior:* 9-15 /group *Offspring brain measures*: not stated  **Exposure Duration:** 2 weeks premating to PND 90-110  **Age at test:** preweaning; PND 30-112 | **Dye Name**:  Red No. 40  **Purity Level:** Not provided  **Dye Source:** H. Kohnstamm & Co  **Additional Variable:** Hydroyurea (positive control**)** | **Route of Administration**: Diet  **Doses**: 0%, 2.5%, 5% and 10% diet  **Control**: 0% diet | Preweaning development (surface righting, pivoting, cliff avoidance, negative geotaxis, auditory startle, swimming ontogeny, open field)  M-Maze  Passive Avoidance  Running Wheel  Rotorod  Active avoidance  Postweaning Open Field  Brain measurements: Brain region weights | Preweaning development:  ↓ swimming direction:  at 2.5% diet, PND 6  ↓ swimming paddling at 2.5% diet, PND 6  Passive Avoidance  ↓ retention performance (re-entry latencies) at 2.5% diet, PND 110-112  Postweaning Open-field  Males: ↑ ambulation central section at 5% diet  ↑ rearing on day 3 at 5% and 10% diet,  PND 41-43  Running wheel ↓ running wheel (nocturnal) activity at 2.5%, 5% and 10% diet, PND 30-50 | Brain measurements:  ↓ brainstem weight at 5% diet  ↓ cerebellum weight at 2.5 %, 5% and 10% diet |
| **Reference:** (Tanaka 1994)  **Institution:** Tokyo Metropolitan Research Laboratory of Public Health  **Funding Source:** Not provided  **Ethical Statement**: Not provided  **Conflict of Interest**: Not provided | **Species**: Mice; CD-1  **Sex**: Male and female  **Group Size:** Offspring: 8-9 sex/group  **Exposure Duration:** 4 weeks premating parents to PND 63 offspring  **Age at test:**  Offspring: PND 4-63 | **Dye Name**:  Red No. 40  **Purity Level:**>85%  **Dye Source:** Tokyo Kasei Co. | **Route of Administration**: Diet  **Doses**: 0%, 0.42%, 0.84% and 1.68% diet  **Control**: 0% diet | Preweaning development (surface righting, negative geotaxis, cliff avoidance, swimming behavior, olfactory orientation)  Activity  Water maze | Maze learning:  Males: ↓ time taken on 3^rd^ trial compared to 1^st^ trial at 1.68% diet (within group comparison)  Females: ↓ time taken on 2^nd^  trial compared to 1^st^ trial at 0.42% diet; ↓ time taken on 3^rd^ trial compared to 1^st^ trial at 1.68% diet (within group comparisons) | No brain assessments |
| **Reference**: (Tanaka 1996)  **Institution:** Tokyo Metropolitan Research Laboratory of Public Health  **Funding Source:** Not provided  **Ethical Statement**: Not provided  **Conflict of Interest**: Not provided | **Species**: Mice; CD-1  **Sex**: Male and female  **Group Size:**  *Parents*: 10/sex/group  *Offspring*:  7-10/sex/group  **Exposure Duration:** 4 weeks premating parents to PND 63 offspring  **Age at test:** Parents: PND 56;  Offspring: PND 4-63 | **Dye Name**:  Yellow No. 6   **Purity Level:** >85%  **Dye Source:** Tokyo Kasei Co. | **Route of Administration**: Diet  **Doses**: 0%, 0.15%, 0.30% and 0.60% diet  **Control**: 0% diet | *Parents:* Activity  *Offspring:* Preweaning development (surface righting, negative geotaxis, cliff avoidance, swimming behavior, olfactory orientation)  Activity  Water Maze | *Offspring*: Preweaning development:  Males: ↓ surface righting at 0.30% diet, PND 7; ↓ negative geotaxis at 0.30%, 0.60% diet, PND 4; ↓ swimming direction at 0.30% and 0.60 % diet, PND 4  Females: ↓ swimming direction at 0.15%, 0.30% and 0.60% diet, PND 4; ↓ swimming head angle at 0.30% and 0.60% diet with dose-related trend  Maze learning:  Males: ↓ time taken on 2^nd^ and 3^rd^ trial compared to 1^st^ trial in control group and 0.60% diet group (within and group comparisons)  ↓ number of errors on 3^rd^ trial in the control group compared to the 1^st^ trial (within group comparison) and ↓ number of errors on 3^rd^ trial at 0.60% diet compared to controls (between group comparison)  Females: ↓ time taken on 2^nd^ trial at 0.60% diet and on 3^rd^ trial at 0.15% and 0.30% diet compared to 1^st^ trial group (within group comparison)  ↓ time taken on 2^nd^ trial at 0.15%, 0.30% and 0.60% diet and on 3^rd^ trial at 0.15% diet compared to control group (between group comparison)  ↓ number of errors on 2^nd^ trial at 0.15% and 0.60% diet compared to control group (between group comparison) | No brain assessments |
| **Reference:** (Tanaka 2001)  **Institution:** Tokyo Metropolitan Research Laboratory of Public Health  **Funding Source:** Not provided  **Ethical Statement**: Not provided  **Conflict of Interest**: Not provided | **Species**: Mice; CD-1  **Sex**: Male and female  **Group Size:**  *Parents:* 10/sex/group  *Offspring:*  8-10/sex/group  **Exposure Duration:** 4 weeks premating parents to PND 63 offspring  **Age at test:**  *Parents*: PND 56  *Offspring*: PND 4- 63 | **Dye Name**:  Red No. 3  **Purity Level:** >85%  **Dye Source:** Tokyo Kasei Co. | **Route of Administration**:  Diet  **Doses**: 0%, 0.005%, 0.015% and 0.045% diet  **Control**: 0% diet | *Parents***:**  Activity  *Offspring:*  Preweaning development (surface righting, negative geotaxis, cliff avoidance, swimming behavior; olfactory orientation)  Activity  Water Maze | Activity:  *Parents***:** Females: ↑ number of turns at 0.045% diet (compared to controls; dose related trend)  Activity**:**  *Offspring:* Males: ↓ horizontal activity at 0.045% diet, PND 21 (compared to controls; dose-related trend); ↑ total distance, PND 21 (dose related trend); ↑ in average distance, PND 21 (compared to controls; dose-related trend)  Females: ↑ movements and average distance at 0.045% diet, PND 56 (dose-related trend); ↑ movement time, average speed, total distance at 0.045% diet, PND 56 (compared to controls, dose-related trend) | No brain assessments |
| **Reference:** (Tanaka 2006)  **Institution:** Tokyo Metropolitan Research Laboratory of Public Health  **Funding Source:** Not provided  **Ethical Statement**: Not provided  **Conflict of Interest**: Not provided | **Species**: Mice; CD-1  **Sex**: Male and female  **Group Size:**  *Parents:* 10/sex/group  *Offspring:*  7-10/group  **Exposure Duration:** 4 weeks premating parents to PND 63 offspring  **Age at test:**  *Parents*: PND 56;  *Offspring*: PND 4-PND 63 | **Dye Name**:  Yellow No. 5  **Purity Level:** >85%  **Dye Source:** Tokyo Kasei Co. | **Route of Administration**: Diet  **Doses**: 0%, 0.05%, 0.15% and 0.45% diet  **Control**: 0% diet | *Parents*  Activity  *Offspring:* Preweaning development (surface righting, negative geotaxis, cliff avoidance, swimming behavior, olfactory orientation)  Activity  Water Maze | *Parents*: Males: ↑ vertical activity at 0.15% diet  *Offspring*  Preweaning development:  Males: ↑ surface righting at 0.45% diet, PND 4 ↑ surface righting, PND 4 (dose-related trend test) ↑ cliff avoidance at 0.15% diet, PND 7  Females: ↓ negative geotaxis at 0.45% diet, PND 4 Activity: Males: ↓ movements (dose-related trend), PND 21  Maze learning:  Males: ↓ time taken on 2^nd^ trials compared to 1^st^ trial in controls and at 0.45% diet (within group comparisons)  ↓ time taken on 3^rd^ trial compared to 1^st^ trial at 0.05% diet (within group comparisons)  Females: ↓ time taken on 3^rd^ trial at 0.45% diet compared to 1^st^ trial (within group comparisons)  ↓ number of errors on 3^rd^ trial at 0.45% diet compared to 1^st^ trial, (within group comparison) | No brain assessments |
| **Reference:** (Tanaka et al. 2008)  **Institution:** Tokyo Metropolitan Research Laboratory of Public Health  **Funding Source:** Not provided  **Ethical Statement**: Not provided  **Conflict of Interest**: None | **Species**: Mice; CD-1  **Sex**: Male and female  **Group Size:**  *Parents*: F0, F1: 10/sex/group  *Offspring:*  F1 7-8/sex/group F2 8-9/sex/group  **Exposure Duration:**  4 weeks premating F0 parents to PND 63; F2 offspring (3 generation study)  **Age at test:**  *Parents*: PND 56  *Offspring*: PND 4-PND 63 | **Dye Name**:  Yellow No. 5  **Purity Level:** >85%  **Dye Source:** Tokyo Kasei Co. | **Route of Administration**: Diet  **Doses**: 0.05%, 0.15% and 0.45% diet  **Control**: 0% diet | *Parents F0*  Activity  *Offspring: F1* Preweaning development: (surface righting, negative geotaxis, cliff avoidance, swimming behavior, olfactory orientation)  Activity  Water Maze  *Parents F1* Activity  *Offspring F2* Preweaning development:  (surface righting, negative geotaxis, cliff avoidance, swimming behavior, olfactory orientation)  Activity  Water Maze | *Offspring:F1* Preweaning development:  Females: ↓ surface righting at 0.15% diet, PND 7 ↓ surface righting, PND 7 (dose-related trend)  Males: ↑ swimming direction at 0.15% diet, PND 7  ↑ swimming direction, PND 7 (dose-related trend)  Activity: Males: ↓ move time, total distance, average distance, turns, PND 21 (dose-related trends)  *Offspring: F2* Preweaning development: Females: ↑ surface righting at 0.15% diet, PND 7  Males: ↑ swimming direction at 0.45% diet, PND 7  ↑ olfactory orientation at 0.15% and 0.45% diet, PND 14  ↑ olfactory orientation  PND 14 (dose-related trend)  Activity: Males: ↓ total distance, average distance, average speed, number of turns PND 21 (dose-related trends) ↓ total distance, average distance, average speed, vertical activity PND 56 (dose-related trends) | No brain assessments |
| **Reference:** (Dalal and Poddar 2009)  **Institution**: University of Calcutta  **Funding Source:** Indian Council of Medical Research, New Delhi India; University Grants Commission, New Delhi, India and University of Calcutta, Kolkata, India.  **Ethical Statement**: Provided  **Conflict of Interest**: Not provided | **Species**: Rats, Charles Foster  **Sex**: Male  **Group Size:** Behavioral: 8-12/group  Neurobiochemical parameters: 4-6/group  pargyline: 12/group  MAOIs (clorgyline/ deprenyl): 8-12/group  **Exposure Duration:** 1 dose  **Age at test:** Young adult | **Dye Name**:  Red No. 3  **Purity Level:** 90%  **Dye Source:** Sigma Chemicals Co. | **Route of Administration**: Gavage  **Doses**: 0, 1, 10, 100 and 200 mg/kg  **Control**: Vehicle, distilled water or saline  **Additional variables**: Monoamine oxidase (MAOA) inhibitors: pargyline, 75 mg/kg, i.p. clorgyline, 5 mg/kg, i.p deprenyl, 5 mg/kg, i.p. | Activity (vertical motor activity (rearing))  Brain  Neurobiochemical Measures:  Steady-state levels of 5-HT, 5-HIAA in brain regions (medulla-pons, hypothalamus, hippocampus, and corpus striatum)  MAOA activity  [^3^H]5-HT binding  Accumulation rate of 5-HT and declination rate of 5-HIAA in brain regions  [^3^H]5-HT receptor binding assay in brain regions | Activity:  ↓ vertical motor activity 10, 100 and 200 mg/kg maximal 2 h after exposure and gradually restored by 9 h. Dose-related pattern. Injection of clorgyline and deprenyl 10 min after Red No. 3 counteracted motor activity suppression of Red No. 3 at 100 mg/kg  Injection of clorgyline and deprenyl 10 min after Red No. 3 prevented Red No. 3 suppression of motor activity at 2 h in a dose-related pattern | Brain Neurobiochemical Measures:  ↓ steady-state level 5H-T at 10 ,100 and 200 mg/kg in medulla-pons, hypothalamus and hippocampus 2 h after exposure  ↑ steady-state levels of 5-HIAA in 10, 100 and 200 mg/kg. in hippocampus 2 h after exposure  ↑ MAO-A activity in hippocampus 2 h after exposure  ↓ pargyline-induced 5-HT accumulation rate 2 h after Red No. 3 exposure in medulla-pons at 100 and 200 mg/kg and in hypothalamus at 10, 100 and 200 mg/kg  ↓ in specific [^3^H]5-HT receptor binding 2 h after Red No. 3 exposure in medulla-pons, and hippocampus at 100 and 200 mg/kg and in hypothalamus at 10, 100 and 200 mg/kg |
| **Reference:** (Dalal and Poddar 2010)  **Institution**: University of Calcutta  **Funding Source:** Indian Council of Medical Research, New Delhi India; University Grants Commission, New Delhi, India and University of Calcutta, Kolkata, India.  **Ethical Statement**: Provided  **Conflict of Interest**: No conflict of interest | **Species**:  Rats, Charles Foster  **Sex**: Male  **Group Size:**  Behavioral: 8-12/group  Neurobiochemical :4-6/group  Pargyline interaction: 4-6/group  **Exposure Duration:** 15 or 30 consecutive days  **Age at test:** 12-14 weeks; adult | **Dye Name**:  Red No. 3  **Purity Level:** 90%  **Dye Source:** Sigma Chemicals Co. | **Route of Administration**: Gavage  **Doses**: 0,1,10 and 100 mg/kg  **Control**: Vehicle: distilled water  **Additional variable:** Monoamine oxidase A (MAOA) inhibitor pargyline 75 mg/kg i.p**.** | Activity (vertical motor activity (rearing))  Brain  Neurobiochemical Measures:  Steady-state levels of 5-HT, 5-HIAA in brain regions (medulla-pons, hypothalamus, hippocampus, and corpus striatum)  MAOA activity  Pargyline-induced accumulation rate of 5-HT and declination rate of 5-HIAA in brain regions  Plasma corticosterone | Activity:  ↑ vertical motor activity at 10 and 100 mg/kg, 15 or 30 consecutive days, maximum 2 h after last Red No. 4 administration and gradually restored by 9 h. | Neurobiochemical Measure:  ↑ brain regional (medulla-pons, hypothalamus, hippocampus, corpus striatum) steady-state levels of 5-HT at 10 and 100, mg/kg for 15 and 30 consecutive days 2 h after last administration  ↓ MAOA activity at 100 mg/kg, 30 consecutive days in all brain regions 2 h after last administration  ↑ pargyline-induced 5-HT accumulation in all brain regions, 10 and 100 mg/kg, 15 and 30 consecutive days 2 h after last administration  ↑ plasma corticosterone at 10 and 100 mg/kg,15 and 30 consecutive days, 2 h after last administration  ↑ pargyline-induced increase in corticosterone at 10 and 100 mg/kg, 15 and 30 consecutive days |
| **Reference:** (Gao et al. 2011)  **Institution**: Kartal Education and Research Hospital, Department of Pathology  **Funding Source:** Shandong Luye Research  and Development for Natural Drugs Co. Ltd  **Ethical Statement**: Provided  **Conflict of Interest**: Not provided | **Species**:  Rats, Sprague-Dawley; Mice, KunMing  **Sex**: Male and female  **Weight**: *Mice*: 20 g; *Rats:* 70 g  **Group Size:**  *Mice* 10/group; *Rats* 10/group  **Exposure Duration:** 30 days  **Age at test:** Not provided | **Dye Name**:  Yellow No. 5  **Purity Level:** >85%  **Dye Source:** Guangzhou Sanxiong Food Trading Co. | **Route of Administration**: Gavage  **Doses**:  *Rats*: 0, 125, 250 and 500 mg/kg/d  *Mice*: 0, 175, 350 and 700 mg/kg/d  **Control**: 0 mg/kg/d | *Mice:* Morris water maze  *Mice*: Step-through avoidance  *Rats*: Open-field test  *Rats*: Brain measurements  (oxidative stress, histopathology) catalase, glutathione (GSH-Px), superoxide dismutase (SOD) malondialdehyde (MDA) | *Mice:* Morris Water Maze:  ↑ escape latency on day 5 and 6 at 350 mg/kg/d and on day 4, 5 and 6 at 700 mg/kg/d  *Mice:* Step-through:  ↓ step-through latencies at 350 and 700 mg/kg/d  *Rats:* Open-field Test:  ↑ number of squares crossed (horizontal activity) at 250 and 500 mg/kg/d  ↑ rearing (vertical activity) at 250 and 500 mg/kg/d | Rats: Oxidative stress:  ↓ catalase, GSH-Px, SOD at 250 and 500 mg/kg/d  ↑ MDA at 250 and 500 mg/kg/d  Rats: Histopathology  swelling, vacuolar degeneration, karyopyknosis, nucleoli disappearance and characteristics of apoptosis at 500 mg/kg/d (descriptive) |
| **Reference:** (Tanaka et al. 2012)  **Institution:** Tokyo Metropolitan Research Laboratory of Public Health  **Funding Source:** Not provided  **Ethical Statement**: Provided  **Conflict of Interest**: No conflict of interest | **Species**:  Mice; CD-1  **Sex**: Male and female  **Group Size:** *Parents*: 10/sex/group  *Offspring*: 8-9/sex/group  **Exposure Duration:**  4 weeks premating parents to PND 63 offspring  **Age at test:**  *Parents*: PND 56  *Offspring*: PND 4-PND 6 | **Dye Name**:  Blue No. 1  **Purity Level:** >85%  **Dye Source:** Tokyo Kasei Co. | **Route of Administration**: Diet  **Doses**: 0%, 0.08%, 0.24% and 0.72% diet  **Control**: 0% diet | *Parents* Activity  *Offspring* Preweaning development: (surface righting, negative geotaxis, cliff avoidance, swimming behavior, olfactory orientation)  Activity Extended activity  Maze learning | *Parents*  Activity:  Male: ↑ horizontal activity at 0.08% diet  Female: ↑ move time, ↓ average rear time (dose-related trends)  *Offspring:* Preweaning development: Males: ↓ surface righting at 0.72 % diet, PND 4;  ↓ surface righting, PND 4 (dose-related trend); ↑ negative geotaxis at 0.08% diet, PND 7  Females: ↓ surface righting at 0.72% diet, PND 4  ↓ surface righting, PND 4 (dose-related trend)  ↑ swimming direction at 0.24% diet, PND 7  Activity: Males: ↓ rearing  at 0.08% diet, PND 56  Females: ↓ horizontal activity PND 56 (dose-related trend)   Extended Activity: Males: ↓ horizontal activity at 30 min at 0.08% diet ↑ average rearing time at 10, 20, and 50 min at 0.24% diet   Females: ↓ total distance, average speed, average time of movement at 0.72% diet  Maze learning: Females: ↓ time taken at 0.24% and 0.72% diet  Males: ↓ time taken on 2^nd^ and 3^rd^ trial compared to 1^st^ trial at 0.08% diet (within group comparison)  Females:↓ time taken on 2^nd^ and 3^rd^ trial compared to 1^st^ trial at 0.08% diet and 0.24% diet (within group comparison) ↓ error on 2^nd^ trial compared to 1^st^ trial (within group comparisons) | No brain assessments |
| **Reference:** (Rafati et al. 2017)  **Institution:** Shiraz University of Medical Sciences  **Funding Source:** Grant 94-7521 from Shiraz University of Medical Sciences  **Ethical Statement**: Provided  **Conflict of Interest:** No conflict of interest | **Species**: Rats, Sprague-Dawley  **Sex**: Male  **Initial weight**: 250-280 g  **Group Size:**  *Behavior:* 10/ group *Brain* *measurements*: 6/group  **Exposure Duration:** 7 weeks  **Age at test:** Not stated; young adult | **Dye Name**:  Yellow No. 5  **Purity Level:** Not provided  **Dye Source:** Sigma-Aldrich  **Additional variable:** Vitamin E; antioxidant; 100 mg/kg/d | **Route of Administration**: Gavage  **Doses**: 0, 5 and 50 mg/kg/d  **Control**: Vehicle distilled water | Novel Object Recognition  Eight-arm radial maze  Brain Measurements Medial Prefrontal Cortex (mPFC) and subregions: volume, number of neurons and glial cells, dendrite length, spine density and morphology) | Novel Object Recognition:  ↓ exploration time at 50 mg/kg; addition of vitamin E increased exploration at 50 mg/kg/d  Eight-arm radial maze:  ↑ days to criterion for combined 5 and 50 mg/kg/d groups. Vitamin E decreased days to criterion of 5 and 50 mg/kg/d combined groups  ↑ working and reference memory errors during learning, combined 5 and 50 mg/kg/d groups. Vitamin E led to fewer errors at 5 and 50 mg/kg/d during acquisition phase  ↑ working and reference memory errors at 5 and 50 mg/kg/d combined during retention test. Vitamin E led to fewer errors at 5 and 50 mg/kg/d during retention | mPFC volume:  ↓ total volume at 50 mg/kg/d. Vitamin E prevented cell loss.  Number of neurons and glial cells:  ↓ 50 mg/kg/d. Vitamin E prevented cell loss   Dendrites length: ↓ at 5 and 50 mg/kg/d. Vitamin E prevented cell loss  Spine density and morphology:  ↓ 5 and 50 mg/kg/d. Vitamin E prevented dendritic spine effects |
| **Reference:** (Noorafshan et al. 2018)  **Institution:** Shiraz University of Medical Sciences  **Funding Source:** Grant 94-01-01-9729 from Shiraz University of Medical Sciences  **Ethical Statement**: Provided  **Conflict of Interest**: No conflict of interest | **Species**: Rats, Sprague-Dawley  **Sex**: Female  **Age**: 8 weeks of age  **Group Size:**  *Behavior:*  10/group *Brain*: 6/group  **Exposure Duration:**  6 weeks  **Age at test:**  *Behavior:* 12-14 weeks of age *Brain:* 14 weeks of age | **Dye Name**:  Red No. 40  **Purity Level:** 99%  **Dye Source:** Sigma-Aldrich  **Additional Variable**:  Taurine (anti-oxidant, anti-inflammatory) 200 mg/kg/d | **Route of Administration**: Gavage  **Doses**: 0, 7 and 70 mg/kg/d  **Control**: Vehicle distilled water | Novel object recognition  Eight-arm radial maze  Brain measurements: (cortex (mPFC and subregions): volume, number of neurons and glial cells, dendrite length, spine density and morphology) | Novel object recognition:  ↓ object exploration and ↓ short– and long-term novelty preference at 70 mg/kg/d; prevented by taurine  Eight-arm radial maze:  ↑ working and reference memory errors during acquisition phase at 70 mg/kg/d; prevented by taurine  ↑ working and reference memory errors during retention phase at 7 and 70 mg/kg/d; prevented by taurine | mPFC volume:  ↓ total volume and volume of subregions at 70 mg/kg/d; prevented by taurine  Number of neurons and glial cells:  ↓ number at 70 mg/kg/d; prevented by taurine  Dendrite length:  ↓ length at 70 mg/kg/d; prevented by taurine  Morphology and density of dendritic spines: ↓ density at 70 mg/kg/d; prevented by taurine |

##### Table A.6 Dye mixture. Developmental and adolescent/adult studies.

Results columns present statistically significant differences between a dose group and control group reported by authors. Statistically significant dose trend tests reported by the authors are also presented. Arrow (↑/↓) indicates direction of difference from control group. For additional variable measurements, statistically significant differences with dye-treatment exposures are presented. PND=postnatal day.

| **Study Information** | **Experimental Design** | **Dye Information** | **Exposure** | **Outcome Assessed** | **Behavioral Results** | **Brain Measurements Results** |
| --- | --- | --- | --- | --- | --- | --- |
| **Reference:** (Shaywitz et al. 1979)  **Institution**: Pediatric Neurology, Yale University School of Medicine  **Funding Source:** NIH grant: Nutrition Foundation  **Ethical Statement**: Not provided  **Conflict of Interest**: Not provided | **Species**: Rats, Sprague Dawley  **Sex**: Male and female (equal number in each group)  **Group Size:** 19-20/group (spit litter design)  **Duration of Exposure:** PND 5-33; pups dosed  **Age at test:** PND 12-30; PND 12, 15, 19, 26 (activity); PND 21 (T-maze avoidance); PND 28 (shuttlebox avoidance) | **% of Dye in Nutrition Foundation Mixture**:  Red No. 3 6.0  Blue No. 1 3.12  Blue No. 2 1.70  Green No. 3 0.13  Yellow No. 5 26.91  Yellow No. 6 22.74  Orange B 0.54  *(Note: Red No. 40 % was not stated in paper, but other papers using this mixture gave Red 40 as 40% of mixture)*  **Purity Level:** Not provided  **Dye Source:** H. Kohnstamm, Nutrition Foundation Mixture  **Additional variable:** 6-hydroxydopamine **(**6-OHDA) (model of ADHD) | **Route of Administration**: Gavage  **Doses**: 0, 0.5, 1 and 2 mg/kg/d mix  **Control**: 0% dyes in gavage fluid | Open field  T-maze escape  Shuttle box avoidance  Brain catecholamines  dopamine and norepinephrine | Open Field:  ↑ activity (percent time active) at 2 mg/kg/d compared to 1 mg/kg/d, PND 12,15 and 26  T maze avoidance ↑ escape latency 0.5 mg/kg/d | No dye treatment effects |
| **Reference:** (Goldenring et al. 1980)  **Institution**: Pediatric Neurology, Yale University School of Medicine  **Funding Source:** The Thrasher Research Foundation and the Nutrition Foundation  **Ethical Statement**: Not provided  **Conflict of Interest**: Not provided | **Species**: Rats, Sprague Dawley  **Sex**: Male and female  **Group Size:** Group 1: treatment (7 pups)  Group 2: control (12 pups)  **Duration of Exposure:** PND 4-PND 30   “Pup in a cup:” Pups reared in synthetic environment separated from mother  **Age at test:** PND 12,15, 19 and 26 (activity) 28 (learning) and 30 (brain) | **% of Dye in Nutrition Foundation Mixture**:  Red No. 3 6.0  Red No. 40 38.71  Blue No. 1 3.12  Blue No. 2 1.70  Green No. 3 0.13  Yellow No. 5 26.91  Yellow No. 6 22.74  Orange B 0.54  **Purity Level:** Not stated  **Dye Source:** Nutrition Foundation Mixture  **Additional variable:** 6-hydroxydopamine **(**6-OHDA) (model of ADHD) | **Route of Administration**: infusion via intragastic catheter  **Doses**: 0, and 1 mg/kg/d mix  **Control**: 0% dyes in gavage fluid | Open field activity  Shuttle box avoidance  Brain catecholamines Dopamine and norepinephrine | Activity:  ↑ activity (percent time active) PND 12, 15, 19 and 26  Shuttle box avoidance  ↓ number of avoidances, PND 28 | Brain catecholamines:  No dye treatment effects |
| **Reference:** (Goldenring et al. 1982)  **Institution**: Pediatric Neurology, Yale University School of Medicine  **Funding Source:** The Thrasher Research Foundation and the Nutrition Foundation  **Ethical Statement**: Not provided  **Conflict of Interest**: Not provided | **Species**: Rats, Sprague Dawley  **Sex**: Male and female (equal number in each group)  **Group Size:** 8/group; Group 1: control  Group 2: sulfanilic acid  Group 3: 6-OHDA  Group 4: 6-OHDA and sulfanilic acid  (split litter design)  **Duration of Exposure:** PND 5 to 30  **Age at test:** PND 12,15, 19 and 26 (activity) 28 (learning) and 30 (brain) | **Metabolite of Dyes in Nutrition Foundation Mixture**:  Sulfanilic acid (p-amino-benzoic acid), metabolite of Yellow No. 5 and No. 6  **Purity Level:** 99%  **Dye Source:** Sigma  **Additional variable:** 6-hydroxydopamine **(**6-OHDA) (model of ADHD) | **Route of Administration**: Gavage  **Doses**: 0 and 1 mg/kg/d  **Control**: Saline | Open field activity  T Maze escape  Shuttle box avoidance  Brain catecholamines dopamine and norepinephrine | Activity:  ↑ activity (percent time active) PND 15 26  T maze Escape Performance:  ↑ escape latency PND 21 | Brain catecholamines:  No dye treatment effects |
| **Reference:** (Kantor et al. 1984)  **Institution:** Rutgers University  **Funding Source:** New Jersey Agricultural Experiment Station: General Foods Corporation  **Ethical Statement**: Not provided  **Conflict of Interest**: Not provided | **Species**: Rats, Wistar  **Sex**: Male  **Group Size:** 7-8/group  **Dye Exposure:** PND 24; 9-day baseline + 33 days testing  **Age at test:** PND 24-65 | **% of Dye in Nutrition Foundation Mixture**:  Red No. 3 6.08  Red No. 40 38.96  Blue No. 1 3.12  Blue No. 2 1.70  Green No. 3 0.13  Yellow No. 5 27.09  Yellow No. 6 22.92  **Purity Level:** Not provided  **Dye Source:** Not stated | **Route of Administration**: Diet  **Doses**: 0%, 0.5%, 1%, 2% and 4% mixture in diet  **Control**: 0% diet | Activity: (24 hour stabilimeter cage)  Biochemical parameters:  Neurotransmitters (serotonin, norepinephrine and dopamine)  metabolites (5-hydroxyindoleacetic acid and homovanilic acid)  Tissue pyridoxal phosphate (PLP) | Activity:  ↓ locomotor activity at 4% diet, between 35 and 53 days | No dye treatment effect |
| **Reference:** Reisen and Rothblat, 1986  (Reisen and Rothblat 1986)  **Institution**: Department of Psychology, The George Washington University  **Funding Source:** H. Kohnstamm and Co. (partial supplying food coloring)  **Ethical Statement**: Not provided  **Conflict of Interest**: Not provided | **Species**: Rats, Long-Evans  **Sex**: Male and females  **Group Size:** 10-12  **Exposure Duration:** PND 2 to 65  **Age at test:**  PND 2 until PND 46 | **% of Dye in Nutrition Foundation** **Mixture**:  Red 3 6.0  Red 40 38.71*  Blue 1 3.12  Blue 2 1.7  Green 3 0.13  Yellow 5 26.91  Yellow 6 22.7  Orange B 0.54*  **Note: Two different dye mixtures administered per group. Second dye mixture identical to first except increased amount of Red No. 40 in lieu of Orange B.*  **Purity Level:** Not provided  **Dye Source:** H. Kohnstamm, Nutrition Foundation Mixture | **Route of Administration**:  Gavage  **Doses**: 0, 2 and 5 mg/kg/d mixture  **Control**: Not provided | Rope descent (PND 14 until criterion)  Position discrimination  Open-field  Observational activity measure  Visual discrimination | No dye treatment effects | No brain assessments |
| **Reference:** ^a^(Ceyhan et al. 2013; Doguc et al. 2013)  **Institutions:** Suleyman Demirel University  **Funding Source:** Scientific Research Fund of Suleyman Demirel University  **Ethical Statement**: Provided  **Conflict of Interest:** No conflict of interest | **Species**: Rats, Wistar Han  **Sex**: Male and female  **Group Size:**  *Behavior:*  15 dam/group; offspring  male 10/group female 10/group *Brain:* 15 dam/group; offspring  male 12/group female 12/group  **Exposure Duration:** 1-week premating to birth  **Age at test:** Behavior:  90 days Brain:  90 days | **Dye Mixture(mg/kg/d)**:  Red No. 3 0.1  Red No. 40 7.0  Blue No. 1 12.5  Blue No. 2 5.0  Yellow No. 5 7.5  Yellow No. 6 2.5  Amaranth 0.5  Azorubine 4.0  Ponceau 4R 4.0  **Purity Level:** Not provided  **Dye Source:** Narmacol, India; Roha, India; Neelicon, India;  KRK, Turkey | **Route of Administration**: Gavage  **Dose**: 43.1 mg/kg/d mixture  **Control**: Vehicle, water | Water maze  Porsolt forced swim  Open field (locomotor, exploratory, anxiety-related behavior)  Protein expression Hippocampus neurotransmitter receptor subunits  NR2, NR2B  nAChR𝛼4, nAChR𝛽2 nAChR𝛼7 | Open-field:  Males: ↓ edge duration in dye treatment group compared to control group  Males and females combined:  ↑ number of line crosses and wall rears in dye treatment group compared to control | Protein expression/Hippocampu:  Males: ↑ NR2B, nAChR𝛽2 in dye treatment group compared to control group ↓ nAChR𝛼 in dye treatment group to compared to control group  Females: ↓ NR2B in dye treatment group when compared to control group |
| **Reference:** (Erickson et al. 2014)  **Institutions:** University of Lethbridge  **Funding Source:** Province of Alberta; Canadian Institutes of Health  **Ethical Statement**: Provided  **Conflict of Interest**: None | **Species**: Rats, Long-Evans  **Sex**: Male  **Group Size:** 8/group  **Exposure Duration:** PND 22-50  **Age at test:** Locomotor activity and emotional behaviors at 1.5 months, 3 months, 7 months and 13 months | **Dye Mixture (mg/kg/d):**  Red No. 40 6  Yellow No. 5 6 Yellow No. 6 6  Blue No. 1 6  **Purity Level:**  Not provided  **Dye Source:** Sigma-Aldrich  **Additional variable**:  Maternal stress  (fours generation of stressed dams) | **Route of Administration**:  Drinking water  **Doses**: 24 mg/kg/d dye mix  **Control**: Vehicle, water | Open field  Affective exploration (emergency latency, refuge time, refuge exits) | Open field:  ↑ movement time in dye treatment group compared to control group, 1.5 months  Affective exploration:  ↓ emergence latency in dye treatment group compared to control group, 3 months  ↑ refuge exit in dye treatment group compared to control group, 3 months | No brain measurements |
| **References:** (Doguc et al. 2015)  **Institutions:** Suleyman Demirel University  **Funding Source:** Not provided  **Ethical Statement**: Provided  **Conflict of Interest**: No conflict of interest | **Species**: Rats, Wistar Han  **Sex**: Male and female  **Group Size:** *Dams*: 15 /group dosed; *Offspring*: 8 males and 8 female/group  **Exposure Duration:** 1-week premating to birth  **Age at test:** One month | **Dye Mixture (mg/kg/d)**:  Red No. 3 10  Red No. 40 700  Blue No. 1 600  Blue No. 2 500  Yellow No. 5 750  Yellow No. 6 250  Amaranth 15  Azorubine 400  Ponceau 4R 70  **Purity Level:** Not provided  **Dye Source:** Narmacol, India; Roha, India; KRK, Turkey | **Route of Administration**: Gavage  **Doses:** 3295 mg/kg/d mixture  **Control**: Vehicle, water | Water Maze  Open-field (spontaneous exploratory and locomotor activity and anxiety-related behaviors)  Porsolt forced swim | Water maze:  Sex difference in dye group but not control group: ↑ latency to locate the visible platform in females  Open-field:  Males and females combined: ↑ wall rears in dye treatment group compared to control group  Porsolt forced swim:  Females: ↑ mobility and ↓ immobility periods in dye treatment group compared to control group | No brain measurements |
| **Reference:** (Doguc et al. 2019)  **Institution:** Suleyman Demirel University  **Funding Source:** Suleyman Demirel University (grant number 3110-TU-12)  **Ethical Statement**: Provided  **Conflict of Interest**: No conflict of interest | **Species**: Rats, Wistar albino  **Sex**: Male and female  **Group Size:**  *Dams*: 15/group   *Offspring*:12/group  **Exposure Duration:** 1-week premating to birth  **Age at test:**   3 months | **Dye Mixture (mg/kg/d)**:  Red No. 3 10  Red No. 40 700  Blue No. 1 600  Blue No. 2 500  Yellow No. 5 750  Yellow No. 6 250  Amaranth 15  Azorubine 400  Ponceau 4R 70  **Purity Level:** Not provided  **Dye Source:** Not provided | **Route of Administration**: Gavage  **Doses:** 3295 mg/kg/d mixture  **Control**: Vehicle, water | Water maze  Open-field (locomotor, exploratory, anxiety-related behavior)  Forced swim  Protein expression  Hippocampus neurotransmitter receptor subunits  NR2A, NR2B  α7 nAChR | Water maze:  ↓ swim speed, visible trial, in dye treatment group compared to control group  Open-field:  Males: ↑ time in inner and central zones ↓ time in outer zone compared to control group  Forced swim  ↓ mobility time and ↑ immobility time in the dye treatment group (males and females combined) compared to the control group | Protein expression  Hippocampus  Females: ↓ NR2A and NR2B in dye treatment group compared to the control group |

^a^Doguc et al., 2013 and Ceyhan et al., 2013 are both from the same research unit and the same experimental design was used in both of the published papers.
